# Elemental Characterization of Ciders and Other Low-Percentage Alcoholic Beverages Available on the Polish Market

**DOI:** 10.3390/molecules26082186

**Published:** 2021-04-10

**Authors:** Magdalena Gajek, Aleksandra Pawlaczyk, Piotr Wysocki, Malgorzata I. Szynkowska-Jozwik

**Affiliations:** Faculty of Chemistry, Institute of General and Ecological Chemistry, Lodz University of Technology, Zeromskiego 116, 90-924 Lodz, Poland; aleksandra.pawlaczyk@p.lodz.pl (A.P.); piotr.wysocki@p.lodz.pl (P.W.); malgorzata.szynkowska@p.lodz.pl (M.I.S.-J.)

**Keywords:** ciders samples, beverages, multielemental analysis, trace elements, ICP-MS, ICP-OES, PCA

## Abstract

Seventy-three samples of alcoholic beverages and juices that were purchased on the Polish market and home-made were analyzed for their elemental profiles. The levels of 23 metals were determined by ICP-MS (Ag, Ba, Bi, Cd, Co, Cr, Li, Mn, Ni, Pb, Sr and Tl), ICP-OES (Al, B, Ca, Cu, Fe, K, Mg, Na, Ti and Zn) and CVAAS (Hg) techniques in twenty-five samples of ciders widely available on the Polish market; six samples of home-made ciders; two samples of juices used in the production of these ciders; and forty samples of low-percentage, flavored alcoholic beverages based on beer. The gathered analytical data confirmed that the final elemental fingerprint of a product is affected by the elemental fingerprint of the ingredients used (apple variety) as well as the technology and equipment used by the producer, and in the case of commercial ciders, also the impact of type of the packaging used was proven. These factors are specific to each producer and the influence of the mentioned above parameters was revealed as a result of the performed analysis. Additionally, the inclusion of the home-made ciders in the data set helped us to understand the potential origin of some elements, from the raw materials to the final products. The applied statistical tests revealed (Kruskal–Wallis and ANOVA) the existence of statistically significant differences in the concentration of the following metals: Ag, Al, B, Bi, Co, Cr, Cu, Fe, K, Li, Mg, Na, Ni, Ti and Zn in terms of the type of cider origin (commercial and home-made). In turn, for different packaging (can or bottle) within one brand of commercial cider, the existence of statistically significant differences for Cu, Mn and Na was proved. The concentrations of all determined elements in the commercial cider from the Polish market and home-made cider samples can be considered as nontoxic, because the measured levels of elements indicated in the regulations were lower than the allowable limits. Moreover, the obtained results can be treated as preliminary for the potential authentication of products in order to distinguish the home-made (fake) from the authentic products, especially for premium-class alcoholic beverages.

## 1. Introduction

According to the definition, cider is a fermented alcoholic drink made from fresh or concentrated apple juice. Cider is produced by a fermentation process, usually with added yeast by which the consumption of sugar produces ethanol, carbon dioxide and flavor compounds [1]. Nowadays, cider is a very popular alcohol, and its production is widespread, especially on the North American, Asian, European and Australian continents. Therefore, the individual types of ciders may differ significantly, depending on the region or country in which they are produced [2]. In line with Polish legislation, ciders can be made by the alcoholic fermentation of apple juice or diluted apple juice concentrate with the possibility of adding some extra ingredients, such as pear juice in an amount of up to 40% by volume or sugar [3]. Apples and pears used to produce ciders are sources of many phytochemicals and vitamins, which are beneficial for human health. However, it should be noted that many different factors have a decisive influence on the content of both metals and other compounds that define the taste or smell. Among them, the following can be mentioned: variety and the degree of ripeness of fruits used, the conditions under which fruit trees are grown, the processes used to extract fruit juice, the fermentation parameters and species of microorganisms used for the fermentation process, the natural composition of raw materials, the ingredients formed during fermentation, contact with production and storage equipment and contamination from the production environment [4,5,6]. Among the nutrients, except for the vitamins mentioned above, there are macronutrients (e.g., sodium, potassium, magnesium, calcium, phosphorus, chlorine and sulfur) and trace elements, the so-called microelements (e.g., iron, zinc, iodine, fluorine, copper and selenium). These components play an important role in human nutrition, because they are not synthesized in the organism. However, some of these (such as lead or cadmium) can produce toxic effects if they occur at high concentrations [6]. In recent years, many studies have been conducted in which the content of trace elements and stable isotope ratios has been determined and the issues related to the indicators of the origin of food industry products or for the assessment of safety of consumption have been discussed [6,7,8,9,10,11,12,13]. Information on the metal concentration in ciders is important not only to evaluate the potential human health risks of the consumption of this alcohol but also to use the quantitative data to characterize the quality, origin and authenticity of ciders.

The existence of maximum allowable concentration limits for hazardous substances is intended to protect the health of consumers against toxic substances and to ensure the quality of food products from their origin to the final products. Table 1 summarizes the permissible limits for some elements established by the World Health Organization (WHO), the Organization of Vine and Wine (OIV) and Polish legislation. To the best of the authors’ knowledge, there are no standards that would define the permissible limits in ciders; therefore, a comparison with international standards for drinking water and wine, as well as with the regulations provided by national legislation for water, wine and beer, was made.

Many researchers have proven that multielemental analysis can provide information about the relationship between the chemical composition of products such as wine [10,12,14,15], tea [16], fruit juice [17] and the provenance of soil. The determination of this relationship is possible, because metals migrate from rocks to soil and then from the soil to fruit through the plant’s roots. Therefore, the levels of some elements will be directly related to the composition and properties of the soil as well as to the plant uptake processes. Bednarek et al. (2007) [18] assessed the quality of apples grown in Poland, based on the content of heavy metals (Pb, Cd, Ni, Zn, Cu, As and Hg) and linked the obtained results with the factors which may influence the metal content in apples, such as variety of apples; air pollution; agrotechnical treatment; and, in particular, specific soil characteristics. The authors observed no significant correlations between certain soil properties and the content of cadmium, zinc, mercury and arsenic in apples. The content of nickel was to the largest extent (this influence reached 40%) dependent on certain soil properties, such as the content of available magnesium, silt fraction, humus and available potassium. On the other hand, authors stated that the concentration of copper in apples was substantially dependent on the presence of zinc, nickel, available phosphorus and humus in the soil. The presence of lead was correlated with the amount of dust, colloidal clay, nickel and copper. However, some of authors [19] argue that only a few elements, such as Mg, Mn, Cs, Ba or Sr, are directly linked to soil geochemistry. Therefore, only on the basis of these levels of elements on alcoholic beverages the samples can be discriminated according to geographical origin. Other factors that affect the elemental composition of an alcoholic beverage include climatic factors (precipitations) and anthropogenic sources of contamination, such as agricultural practices (fertilizers and pesticides; surface treatment of fruits before harvesting by, e.g., use of calcium chloride possibly containing small amounts of strontium as a skin hardener) and environmental pollution, as well as the production process itself (e.g., filtration process).

The main aim of the research was to carry out the elemental characterization (Ag, Al, B, Ba, Bi, Ca, Cd, Co, Cr, Cu, Fe, Hg, K, Li, Mg, Mn, Na, Ni, Pb, Sr, Ti, Tl and Zn) of the collected alcohol (71) and juice (2) samples. A discriminatory analysis was performed in relation to commercial ciders and home-made products (produced for the purpose of this study). The gathered analytical data helped to distinguish ciders from low-percentage beer alcohols. Moreover, the obtained results allowed the discrimination process of samples in relation to the packaging used within one producer (can or bottle). The presented outcomes can broaden the knowledge in the field of cider fingerprinting, especially that the number of studies with ciders analysis available in the literature is quite limited. Most researchers who decide to discuss the subject of cider analysis focus on examining the following aspects: volatile compounds, sugar content [5], polyphenols content and color [24], stable isotopes ratios [6,19] or different must supplementation with mineral salts during the fermentation process [25]. Thus, this study concentrates on new or rarely discussed issues in the literature, namely, determining the impact of packaging medium on the elemental composition of commercial and home-made products based on the elemental composition. Those results can be also treated as preliminary for the potential authentication of products in order to distinguish the home-made (fake) from the authentic products, especially for premium-class alcoholic beverages.

## 2. Materials and Methods

### 2.1. Samples

In this study, 73 samples of alcoholic beverages and juices were analyzed. The analyzed set of samples consisted of the following: twenty-five samples of ciders widely available on the Polish market; six samples of home-made ciders; two samples of juices used in the production of these ciders; and forty samples of low-percentage, flavored alcoholic beverages based on beer (radler). In the case of low-percentage alcoholic beverages, they were partially produced by local manufacturers and partially came from large international companies. The cider samples used for the study originated from Polish producers. Studied samples varied both in terms of their type (ciders and low-percentage flavored alcoholic beverages), type of productions (commercial and home-made) and type of packaging (can or bottle). The most important information about the sample type as well as the alcohol content is given in Table S4 (Supplementary Materials). The names of ciders and other alcoholic beverages are coded, and the manufacturers’ names are not given in this paper. Basic characteristics of tested samples are included in Table 2.

### 2.2. Samples Preparation and Equipment

#### 2.2.1. ICP MS and ICP OES

Before measurement by ICP MS and ICP OES techniques, appropriate sample preparation was necessary. This included sample degassing (Ultrasound system) and mineralization (Ultrawave system, Milestone, Via Fatebenefratelli, Italy) processes. For this purpose, 4 mL of 65% HNO3 (Baker, Avantor Performance Materials Poland S.A., Gliwice, Poland) was added to 4 mL of the sample. In order to conduct the decomposition process, an ultrasonic washer was applied (Bandelin Sonorex Digitec, Berlin, Germany). In the next step, microwave mineralization was employed. The parameters of the program used for the mineralization of alcohol samples were given by Pawlaczyk et al., 2019 [9]. After the mineralization process, the contents of the tubes were quantitatively transferred to class “A” flasks, 150 µL of an internal standard was added (In; Merck, Warszawa, Poland) and samples were filled with deionized water up to a volume of 25 mL. An ICP-MS analytical technique (Thermo Electron Corporation, X SERIES, East Lyme, CT, USA) was applied to determine and quantify the levels of metals in cider samples, based on the following isotopes ^107^Ag, ^138^Ba, ^209^Bi, ^111^Cd, ^59^Co, ^52^Cr, ^7^Li, ^55^Mn, ^60^Ni, ^208^Pb, ^88^Sr and ^203^Tl. Concentrations of Al (396.152 nm), B (249.773 nm), Ca (393.366 nm), Cu (324.754 nm), K (766.490 nm), Fe (259.92nm), Mg (279.553 nm), Na (589.592 nm), Ti (334.941 nm) and Zn (213.856 nm) were determined by the ICP-OES (Thermo Scientific, ICAP 7000 series, MA, USA). The content of Ca, Mg, Na and K was determined in the radial plasma position, while the levels of Al, B, Cu, Fe, Ti and Zn were assessed in an axial plasma setting.

Three replicates were performed for each alcohol beverage sample for both techniques. The signal stability was monitored based on the intensity of signal for In used as an internal standard. Information about the operating conditions for the multielemental analysis of ciders performed using the ICP-OES and ICP-MS spectrometers is presented in Table 3.

The accuracy of the applied procedure was verified based on the analysis of the certified reference material of TMDA 64 (fortified lake water sample by National Water Research Institute, Burlington, Halton, ON, Canada). The same procedure of verifying the accuracy of the proposed method was described elsewhere by Gajek et al., 2021 [10]. This Certified References Material has the certified values of 21 elements and information values for 7 elements. Due to the fact that the levels of metals which were certified were higher, CRM was diluted a few times (with different dilutions factors) so that the measured concentration of different elements would be comparable with the levels of elements present in the tested samples of ciders. The obtained recoveries were close to 100%.

Every sample was measured 3 times, and the RSD expressed in %, even for elements measured at very low levels, was in the range of 0.01–5.00%.

#### 2.2.2. Mercury Analyzer

In this study, the automatic mercury analyzer MA-3000 (Nippon Instruments Corporation, Tokyo, Japan) was used to determine total mercury content in beverage samples. The analytical procedure was analogous to the one described by Gajek et al. (2021) [24].

#### 2.2.3. pH-Meter

Measurement of pH of tested ciders and beers was performed by the Basic 20 + pH-Meter (CARISON INSTRUMENTS S.A., Barcelona, Spain), which consists of a magnetic stirrer with automatic temperature stabilization. For the correct measurement, a combined electrode, which consists of a glass electrode with a silver chloride electrode placed in one holder, was used. Before the measurement, calibration was performed by using technical buffers at pH 4.01, 7.00 and 9.21 (HACH Company, Düsseldorf, Germany). Measurements were conducted during a one-day analytical cycle, and for all samples and were repeated 3 times. The average result of every sample was taken as the final result. After analyzing 20 samples, calibration was repeated.

### 2.3. Data Analysis

Statistical and chemometric analysis was carried out by STATISTICA 12.5 software (New York, NY, USA). The first step to the correct use of statistical tests for analysis is to specify the type of distribution of the analyzed variables. The Shapiro–Wilk test was used in order to assess the distribution of all samples for the adopted significance level α = 0.05. The Kruskal–Wallis test was used if the null hypothesis of the normal distribution was rejected. The typical application of this test is the examination of the potential statistically important differences between concentrations of chosen variables among various group pairs. If the hypothesis of normal distribution was confirmed, the parametric ANOVA test was used. All the results are presented in boxplot plots, since they can clearly illustrate the differences in the concentrations of various variables in relation to the selected criteria. Principal component analysis was also performed (PCA).

## 3. Results and Discussion

### 3.1. Level of Metals in Analysed Alcoholic Beverages

In this study, the level of 23 elements in 73 samples of beverages (including ciders, apple juice used in ciders production and other low-percentage alcohol) was determined. The concentration of Ag, Ba, Bi, Cd, Co, Cr, Li, Mn, Ni, Pb, Sr and Tl was measured by the ICP-MS technique. The ICP-OES technique was used to assess Al, B, Ca, Cu, Fe, K, Mg, Na, Ti and Zn content. The mercury content was analysed by the CVAAS technique. In the collected data set, some obtained results were below the detection limits. Thus, Ag and Bi were not determined in two samples, Fe was not determined in 10 samples, Pb was not determined in 19 samples and Ti was not determined in 23 samples. In the case of mercury content, all results were below the limit of detection.

The distribution of the studied variables was checked before the statistical analysis of the results obtained by the ICP-MS and ICP-OES techniques. On the basis of the Shapiro–Wilk tests, the hypothesis on the normal distribution for most of studied variables was rejected. However, elements such as Al, B and Mn showed a normal distribution. Therefore, the ANOVA test was used for these elements. In other cases, a nonparametric test—the Kruskal–Wallis test—was used to analyze the data set.

The statistical information on the studied variables, such as the mean, median, minimum and maximum or standard deviation values, is given in Table 4 and Figure S1 (Supplementary Materials).

Despite the quality control of food products prior to their introduction to the market, the literature report survey clearly indicates that the permissible standards can be exceeded. Using the example of low-percentage alcoholic beverages produced as a result of fermentation, such as wines, several cases of exceeding the permissible norms for metal content can be given. According to the comparison of the content of certain elements in wines from different countries presented by Woldemariam et al., 2011 [26], the limits for cadmium were exceeded in the case of wine from Spain (max content 19 µg/L) and Hungary (max content 54 µg/L), whilst for lead, the maximum permitted content with regard to the OIV standard was exceeded for wines from the Czech Republic (max content 1253 µg/L), Italy (max content 350 µg/L) and Ethiopia (max content 310 µg/L). Some authors have suggested that higher concentrations of these metals are probably the result of the infractions of technological norms, possible pollution from the container or as a consequence of mixing different kinds of grape harvest [27].

In our study, contents of elements such as Al, B, Cr Mn and Ni were higher than the maximum concentration permitted according to the WHO (Al, Cr and Ni) and Polish legislation (B, Cr Ni and Mn). Most of the tested samples exceeded the allowable values, except chromium, where the maximum content was exceeded only in one case. However, it should be emphasized that the acceptable limits for these elements were for drinking water. In turn, for elements such as Cd and Pb, for which the permissible values have been established in both drinking water and alcohols, the maximum obtained results exceeded only standards for drinking water for both elements. In the case of cadmium, the limit for drinking water, which was exceeded only once, was related to the most restrictive standard provided by the WHO (3 µg/L). For lead, where both Polish legislation and WHO standards for water assume the maximum content at the level of 10 µg/L, an exceedance of 45 cases was noted. The limit values were not exceeded in any case for the other elements (Ag, Ba, Cu, Fe, Hg, Mg, Na and Zn). It should be noted that there are no regulations regarding the permissible concentrations for the rest of the determined elements in this study, namely, Bi, Ca, Co, K, Li, Sr, Ti and Tl.

Table 5 compares the ranges of results obtained in this study with the results of other researchers, where elemental analysis was also carried out for ciders [6,19] and beers [13,26]. In the case of the results for ciders, for most of the determined elements (Al, B, Ba, Bi, Cd, Cr, Ca, K, Mg, Na, Li, Mn, Ni, Pb, Ti, Tl and Zn), full compliance was obtained with ranges given by García-Ruiz et al. (2007) [19] and Cristea et al. (2019) [6]. 

Higher levels of Co and Fe determined in this study were found when compared to the literature reports [6,19]. These differences were observed within one cider producer (CMJP and CMPP), which can be treated as the characteristic signature of a particular manufacturer. In turn, the higher content of Cu and Sr, compared to the work of other researchers, was determined in home-made ciders.

In the tested beer-based low percentage aromatic alcoholic beverage samples, elements such as Cd, Co, Cr, Cu, Fe, Mn, Pb, Sr and Zn showed relatively high compliance with the works of other researchers [13,28]. The levels of Ba, Ni and Al determined in this study were higher than those found in the literature, and in some cases, even the standards available for drinking water provided by WHO were exceeded. However, due to the lack of available standards for low-percentage alcohols, it cannot be concluded that the analyzed samples are not safe for the consumers.

Ag and Hg contents in ciders samples were not given by any of the mentioned researchers.

#### 3.1.1. Results Collected for One Brand of Cider—The Influence of Type of Packaging

In order to verify the hypothesis on the potential impact of packaging on the elemental composition of alcohol, we chose the most popular domestic brand of cider and purchased six products from each type of packaging (six samples in a can and six samples in a bottle). The purchased samples in the bottles and cans came from one production batch and were opened at the same time.

Considering the tested set of samples in terms of the type of packaging of the ciders, the existence of statistically significant differences based on Kruskal–Wallis and ANOVA tests in the concentrations of the following elements was found: Cu (*p* = 0.036), Mn (*p* = 0.037) and Na (*p* = 0.025).

**Figure 1:**
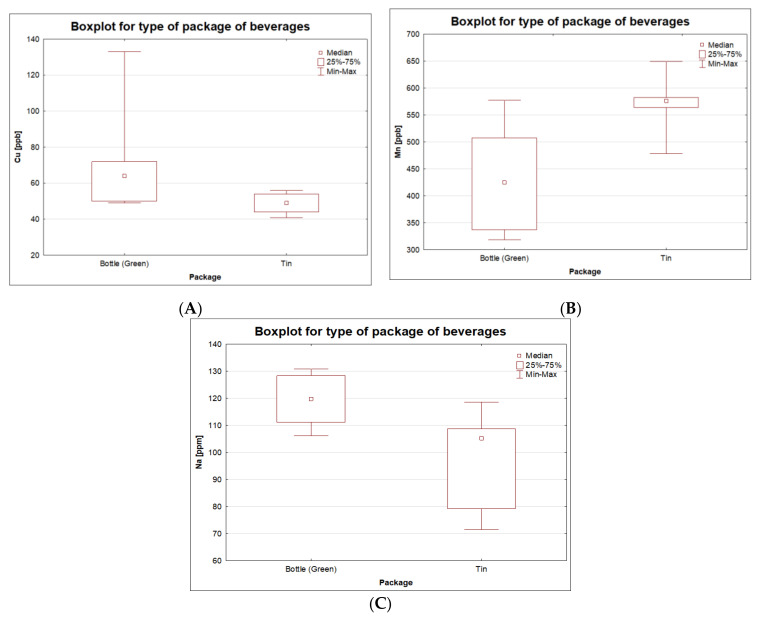
Boxplot for contents of Cu (**A**), Mn (**B**) and Na (**C**) for obtained results of 12 samples of one brand of cider divided according to type of packaging.

Basic information (mean and median value) on Cu, Mn and Na concentrations (µg/L) for ciders stored in bottles and cans is given in Table S2 in the Supplementary Materials and Figure 1A–C.

As expected, the sodium level turned out to be decisive in discriminating cider samples with regard to the packaging in which they were stored. The level of this element was higher in samples from glass bottles. Sodium oxide, in addition to silicon oxide, is among the basic ingredients of glass bottles commonly used in the food industry (it accounts for about 12% of the total composition) [29,30]. It should be emphasized that during the process of sample preparation for analysis (mineralization and dilution), the alcohol samples did not come into contact with glass vessels, which could distort the sodium content. In turn, in the production of food cans, in order to strengthen the resistance to crushing, clean aluminum is combined with harder metals, such as copper or manganese as alloys. The advantage of this solution is the possibility of reducing the weight of the structure [31]. This explains the higher manganese content found in stored ciders. Although copper is also a possible component of aluminum cans, higher levels of this element were obtained in our study for alcohols stored in bottles. This phenomenon can be explained by the fact that only greenish bottles were taken into account for the comparisons of the dependencies of the same cider in relation to the packaging medium. It is likely that in order to obtain a particular shade, the manufacturer used a mixture of chromium and copper compounds, which cannot be excluded by authors as the main reason for which higher copper concentrations were detected in greenish bottles. Moreover, these results were generated by the same brand and manufacturer. This issue connected with the influence of packaging medium on the release of selected elements into the surrounding environment recently is gaining increasing attention [32,33]. The recent literature reports suggest that there is a clear evidence of the existence of a correlation between the packaging medium and the release of various components, including metallic contaminants. Reimann et al., 2010 [33], determined the concentration of 57 elements in 294 samples of bottled water by the ICP-MS technique (where water from the same producers was sold in glass as well as in PET bottles). These authors confirmed a higher concentration of some elements in glass bottles when compared with PET bottles (e.g., Sb, Pb, Zr, Cu, Al, Fe, Ti, Zn, Cr and Sn). Additionally, in the same paper, the influence of the color of glass bottles was investigated, and it was confirmed that water kept in green glass bottles had significantly higher concentrations of elements such as Cr, Ti, Fe and Co. In our study, only six bottles from one producer were examined, while in the work of Reimann et al., 2010, authors examined 136 pairs of cases where water was sold in clear and green bottles by the same and different producers (the majority of them originated from Germany but also from the rest of Europe). It should be noted that the leaching tests carried out showed that the median value for copper was slightly higher for PET bottles as compared to glass bottles (0.243 µg/L PET, 0.226 µg/L glass bottles). In the case of our study, which had a much smaller comparative group (n = six cans and n = six bottles), higher values of copper in alcohol stored in a bottle were obtained. Admittedly, in the case of chromium, the Kruskal–Wallis test showed no statistically significant differences in relation to the packaging; however, as expected, the highest value was recorded for the sample in the glass packaging, and the lowest for the sample stored in the can. The Cr median value was also higher for the bottle sample group. There were also no statistically significant differences in relation to the aluminum content. However, for the alcohol samples taken from the cans, the median value was higher compared to the samples taken from the bottles. This is naturally related to the main component of aluminum cans.

#### 3.1.2. Results Collected for All Analyzed Ciders—Comparison of Commercial Ciders with Home-Made Products

In order to compare the ciders available on the Polish market with home-made products, the authors of this article decided to study home-made alcohol which, according to the definition, complied with all cider requirements. Two different ciders were produced from two different juices. The first cider (CR) was produced from commercial, clarified apple juice (100% juice) made from a mixture of sweet and sour apples from local cultivation (Grójec). The second cider (CL) was made from apple juice directly and independently squeezed from apples of the Ligol variety, also coming from the local cultivation (Skierniewice). The juice was naturally cloudy, not clarified. When choosing products from which the ciders were then made, the authors were guided by the high-quality and local nature of the raw materials. Polish apples have been appreciated by recipients all over the world for many years. It should be underlined that in terms of sales volume, Poland is the largest producer in the EU and the 3rd largest global producer of this fruit [34].

“Ligol” is a variety that was selected at the Institute of Horticulture and Floriculture (now the Research Institute of Horticulture) in Skierniewice in 1972 from the crossing of “Linda” and “Golden Delicious” varieties. Their sweet and sour taste and juicy flesh provide ideal properties for making cider [35].

Three independent analytical samples were taken from each cider. In addition, the juices from which the ciders were produced were also analyzed.

In the next stage of data evaluation, the potential statistical differences in the level of studied elements were tested. For this purpose, Kruskal–Wallis and ANOVA tests were employed. It was revealed that when considering the studied samples in terms of the type of origin (commercial or home-made), the existence of statistically significant differences in the concentration of the following metals could be observed: Ag, Al, B, Bi, Co, Cr, Cu, Fe, K, Li, Mg, Na, Ni, Ti and Zn (Table 6 and Figure S2A–O in Supplementary Materials). In the mentioned cases, the level of significance (*p*) was less than 0.05.

Taking into account the median value for the following elements, this value was higher for home-made ciders compared to commercial ciders: Bi, Co, Cr, Cu, Fe, K, Li, Mg, Ni, Ti and Zn. For Ag, Al, B and Na, the median value was higher in the case of commercial ciders. Home-made cider is distinguished by its much lower sodium content. This is probably related to the fact that during the production process, this alcohol had no contact with any glass vessel. Analyzing the boxplots (Figure S2A–O in Supplementary Materials), the greatest attention is paid to elements such as potassium and nickel. In the case of these elements, much higher and very similar levels within the group of home-made ciders were recorded. This is confirmed by very narrow boxes and very short whiskers (Figure S2I,M in Supplementary Materials).

It should be emphasized that the elemental characteristics of home-made ciders are closely related to the juice from which they are made. Thus, the elemental profile of ciders produced from various juices is determined primarily by the variety of apples used in the ciders; the conditions in which they are matured; and the possible plant protection products used, which is best defined in particular by differences existing between home-made ciders. Despite the uniform method of production, the same yeast and fermentation vessels made from identical materials, the existence of statistically significant differences for two ciders (CL and CR) in the concentrations of the following elements was found: Ba, Ca, Co, Cu, K, Li, Mg, Mn, Na and Sr (Table S1 and Figure S3A–J in Supplementary Materials). For the above-mentioned elements, the differences in the median value were very large within the compared groups. However, only the levels of copper and potassium were higher in the CL cider (cider made from freshly squeezed ligol apple juice). For the remaining elements, higher values were recorded for CRL cider (made from 100% apple juice, bought in a store). However, the most notable difference is in the content of strontium. In the case of CR cider, on average, there is over twenty times more of this element compared to CL cider. For cider samples, the presence of strontium could be related to their provenance soil due to the weathering of rocks and the transference of Sr to the soil–water system and subsequently to the apple [19,36]. This is especially true of the ^87^Sr/^86^Sr isotope abundance ratio. Researchers emphasize that Sr isotope abundance ratios in the ciders could be directly linked to those in the apples and, when the fruits originate from the same geographical area, to their original soil [19]. Strontium isotope abundance ratios were used to investigate the geographical origin of different food and beverage products, such as wine [37], cheese [38], honey and meat [39].

Despite the fact that the cultivations from which the apples were used for the production of both ciders come are located relatively closely (about 100 km), there are many differences in the elemental characteristics between them. This is probably due to different soil conditions or different varieties of apples. The juice for the production of CL cider was made from apples of the ligol variety; however, the authors, unfortunately, do not know exactly which apple varieties were used to make the juice used in the production of CR cider (it was a mixture of different varieties). Therefore, the comparison in this aspect is impossible.

### 3.2. Chemometric Analysis of Multiparametric Data

**Figure 2:**
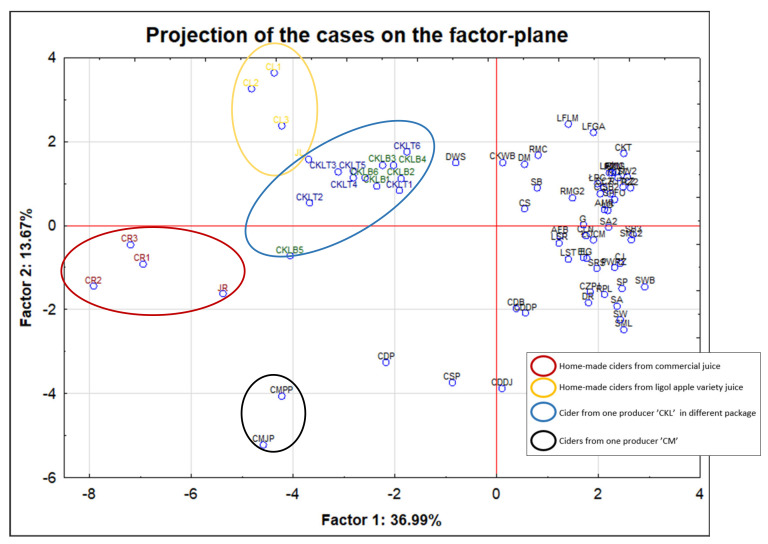
Projection of the cases on the factor-plane in 73 samples investigated in this study according to their producer code.

In this study, authors made a projection of the factor-plane for 73 independent alcohol and juice samples (Figure 2). The projection confirmed earlier conclusions that the characteristics of home-made ciders are closely related to the juice from which they are made. The CR cider (samples from 1 to 3) and JR juice are grouped into one cluster marked in red. The CL cider (samples from 1 to 3) and JL juice sample are grouped in a similar way (marked in yellow). Although there are statistically significant differences for sodium, manganese and copper within different packaging (can CKT1-6 and bottle CKB1-6), cider samples from one producer also form a common cluster.

CMJP and CMPP ciders also come from one cider producer. These samples constitute outliers in the performed projection due to the higher contents of iron and cobalt in relation to other samples. It should be noted that CMJP cider is a classic apple cider. In turn, CMPP is a kind of so-called perry. However, the uniform production process gives the products the same elemental features that make it possible to distinguish them from the others. Furthermore, the mentioned cider is stored in brown bottles, and as commonly known, iron compounds are used to color the glass brown, which may be important in the context of the increased content of this element in these samples. Additionally, it should be noted that the projection of the cases on the factor-plane allowed relative separation of ciders from low-percentage flavored alcoholic beverages. The vast majority of the examined cider samples are found in the third and fourth quarters of the projection, while all (except one case—DWS) beer drinks are located in the first and second quarters. Comparing the results for all samples from Table 5 it can be easily seen that the ciders are characterized by higher contents of the following elements: Mn, Ni, Zn, Fe, K, Sr and Cu. In turn, other low-percentage beverages are richer in Na and Mg. 

In the case of studies dedicated to discrimination analysis (grouping of samples according to selected parameters, such as manufacturer and country of production), the origin of the base product (e.g., variety of apple) for alcohol production can be crucial. We should be aware that many factors can influence the composition of the final product, since the base products can have different accumulation abilities; can be harvested in areas with various pollution degrees, with or without pesticide employment; and can come from different parts of plants (grains vs. fruits). Pepi et al., 2018 [40], established a direct relationship between the chemical elements detected in soil and in plant parts (leaf and strobili samples) from Cavalese and Ime’r (Italy) using X-ray fluorescence and inductively coupled plasma mass spectrometry. It was shown that based on the concentrations of major elements, such as C, Fe, Mg and Na, and trace elements, such as Co, Cu, Ni, Pb and Zr (together with their correlation maps), it was possible to identify soils according to their geographical origin. The authors proved that a reliable dependence between the geolithological features of soil and the chemical composition of strobili, based on the content of Fe, Nb, Rb, Zr for Cavalese and Mg, Ni, Zn and Zr for Ime’r, can be found. In turn, the multivariate analysis by PCA and PLSDA showed that it was possible to determine the geographical origin of samples of soil, leaf and strobili from Cavalese and Ime’r according to the presence of Ca, Cr, Fe, Mg, N, Ni, Ti and Zr. All these obtained dependencies also revealed possible effects of local agricultural practices. These elements turned out to be suitable to establish geochemical fingerprints of hop plants belonging to the local variety Hallertau Perle and grown in the Trentino Region, whose strobili are the main ingredients to produce fine home-brewed Italian beer. In turn, Gramss 2020 [41] carried out a controlled malting and brewing process. At each stage, the concentrations of the selected elements were assessed (from grain to the final product). The resulting transparent barley and wheat beers obtained by the aforementioned author concurred with the guidelines for drinking water and alcoholic beverages. The author compared plants and products resulting from their processing (malt, draff, wort and beer), grown on soil contaminated with heavy metals and on soil free of them. The author proved that the grains grown on soil free from heavy metal contamination showed lower values of these elements than those grown on contaminated soil. With regard to distinguishing different types of cereals (wheat and barley) and products resulting from their processing, on the basis of their elemental characteristics, nickel and chromium can be treated as discriminating metals. In the case of other raw materials used for the production of alcohol fruit, apples should be mentioned. Bednarek et al., 2007 [18], determined the concentration of heavy metals in apples grown in Poland (Pb, Cd, Ni, Zn, Cu, As and Hg). Additionally, authors linked the obtained results with the factors which may influence the metal content in apples, such as the variety of apples; air pollution; agrotechnical treatment; and, in particular, specific soil characteristics. It was noted that the content of nickel was strongly influenced by particular soil properties, such as the content of available magnesium, silt fraction, humus and available potassium. In turn, the presence of lead was correlated with the amount of dust, colloidal clay and levels of nickel and copper. It should be emphasized that the quantitative analysis of selected elements was performed at very low levels. These levels were much lower than in the case of the elemental characterization of wines, conducted in an analogous manner by the authors of this publication, especially with regard to trace elements [10]. This is extremely important in the case of the discriminant analysis of one manufacturer. As shown in Figure 2 (can CKT1-6 and bottle CKB1-6), even within one production batch of ciders stored in cans and bottles, there are differences that do not allow us to clearly confirm the packaging used. When trying to discriminate according to quantitative analysis at such a low level, relative standard deviation (RSD) should be used as an indicator of the minimum scatter of results that can be achieved to determine the interobject difference.

In order to eliminate factors that cause deviations in the test method, water samples (used to produce home-made ciders) were tested as the blank control. The water samples taken directly from the tap were diluted and treated in the same way as the studied samples. In order to test the possible variation in the elemental composition over time, samples were taken twice a day (in the mornings and in the afternoons when differences in water consumption may occur). Additionally, the projection of the cases on the factor-plane, including samples of tap water collected over the course of several days, twice a day, is shown in Figure S4 in the Supplementary Materials. The dispersion within the aforementioned group of water samples was small. Despite the fact that slight differences in the concentration of the selected metals relative to the time of day were noticed, the RSD values for particular elements did not exceed 1%. Therefore, it can be assumed that the dispersion of the results of the determined metals in relation to the day of sampling hardly influences the possibility of distinguishing food products, including ciders, e.g., in terms of the brand. The content of the determined metals in the tap water was mostly at a trace level or below the detection limit, which is in line with the data provided by the local water and sewerage company [42]. In addition, it should be emphasized that the authors only had access to samples of tap water used in the production of home-made ciders manufactured for the purpose of this study (but taken a few weeks after alcohol production). In the case of other commercial ciders produced in Poland, manufacturers use local spring or tap water, the elemental composition of which may vary significantly. However, the authors did not have access to these.

3.3. pH Measurement

All the ciders, juice and other low-percentage alcoholic beverage samples were tested against the pH value, which was used as an additional variable for potential sample discrimination and categorization by the STATISTICA 12.5 software. The basic statistical parameters of the results obtained for all alcoholic samples according to the chosen parameters are presented in Table S3 in the Supplementary Materials.

The normality of the distribution for the pH mean value divided into individual groups was tested. The obtained *p*-value (at significance level of 0.05) implied that for the mean pH of alcohol samples in particular groups, the outcomes do not follow the normal distribution.

**Figure 3:**
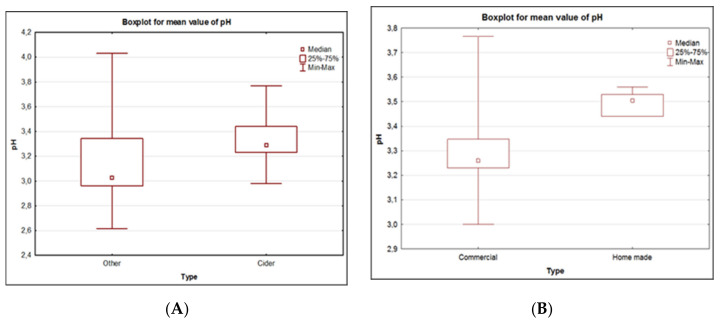

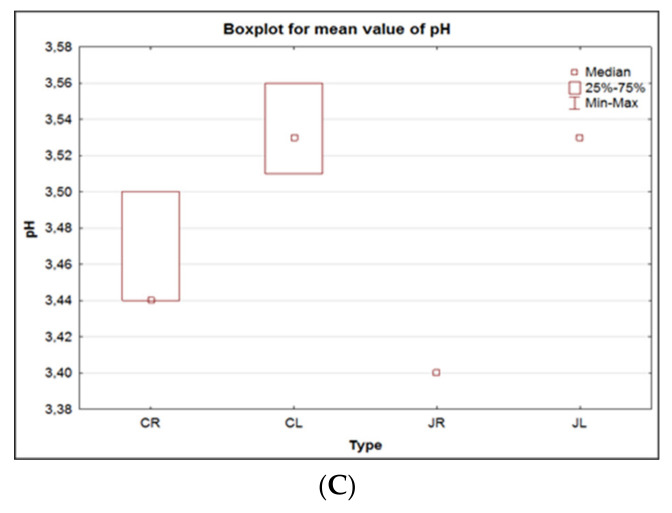
Boxplot of pH value divided according to type of alcohol (ciders–others (**A**)), type of production of ciders (commercial–home-made (**B**)) and type of juice used for home-made ciders production (CR-CL-JR-JL (**C**)).

Comparing the pH median value of ciders and other alcoholic beverages, it can be noticed that in the case of ciders, this value is higher (Figure 3). This is due to the fact that for low-percentage beverages based on beer, producers declare the addition of acidity regulators (citric acid, malic acid, ascorbic acid, etc.). By analyzing only the group of ciders unequivocally on the basis of the median value, it can be concluded that home-made ciders are characterized by higher pH values. This, in turn, is directly related to the pH values measured for the juices from which the ciders were produced. The median value for JL juice is exactly the same as for the cider that was made from it (3.53). On the other hand, the median value for JR juice is only 0.04 lower compared to the produced cider (3.44). The lower pH value for JR juice compared to JL juice can be explained by the fact that the producer declares that the juice contains vitamin C. In turn, JL juice is freshly squeezed from apples and is not modified in any way.

The highest pH values were obtained for juices (JR and JL). The median value in this case was 3.47. For ciders, it was 3.29. The lowest median value was achieved for the other alcoholic beverages—3.03.

In the case of the work by Nicolini et al. (2017) [24], the pH values of ciders made from seven different varieties of apples and a mixture of different varieties were examined. The obtained results were in the range of 3.11–3.61, which is in line with the averaged results obtained in this study for ciders.

## 4. Conclusions

The elemental composition of 73 samples of commercial and home-made ciders, low-percentage beer alcohols and juices was analyzed. All results were chemometrically and statistically processed to establish the most efficient markers that are able to differentiate the type of alcohol and type of packaging used. The final elemental fingerprint of a product is given by the elemental fingerprint of the ingredients used (apple variety) as well as the technology and equipment used by the producer, and in the case of commercial ciders, also the type of packaging used. These factors are specific to each producer, as our performed analysis revealed. The distinction between commercial and home-made ciders was possible to the greatest extent on the basis of the content of Ni and K. In turn, the differences between home-made ciders produced from different juices were most visible for Sr, Cu and K. In the case of commercial cider from one producer and stored in different packaging (can or bottle), as a discriminating criterion, the difference in the content of Na, Cu and Mn should be indicated.

The concentrations of the major and minor elements analyzed in the commercial cider from the Polish market and home-made cider samples can be considered nontoxic, because the content of these elements is significantly lower than the allowable limits.

Moreover, these analyses can be quite interesting from the perspective of people creating their own home-made ciders, as shown in this paper, especially in that the safety of products is not checked before their distribution/consumption. These results can also be treated as preliminary from the authenticity of products point of view, where the direct comparison of original products with possible fake ones is crucial. The possible differentiation of home-made products from commercial ones, as described in this paper, can broaden our knowledge in this field.

Cider is a traditional alcoholic drink, which is gaining increasing popularity in Poland. However, its chemical composition has received little attention. This study contributes towards a better characterization and description of ciders made in Poland.

## Figures and Tables

**Table 1:** The maximum admissible concentration of selected elements established by the Organization of Vine and Wine (OIV) (in wine) [20], the World Health Organization (WHO) (in water) [21] and by Polish legislation (in water [22], beer and wine products [23]).

Origin	Maximum Concentration of Metals [mg/L]														
	Ag	Al	B	Ba	Cd	Cr	Cu	Fe	Hg	Mg	Mn	Na	Ni	Pb	Zn
**WHO (water)**	−	0.9	−	1.3	0.003	0.05	2.00	−	−	−	−	−	0.07	0.01	−
**OIV** **(wine)**	−	−	−	−	0.01	−	1.00	−	−	−	−	−	−	0.15	5.00
**POLISH (water)**	0.01	−	1	−	0.005	0.05	2	0.2	0.001	7–125	0.05	200	0.02	0.01	5.00
**POLISH (beer)**	−	−	−	−	0.02	−	−	−	0.01	−	−	−	−	0.1	−
**POLISH (wine)**	−	−	−	−	0.01	−	1.00	−	0.01	−	−	−	v	0.2	5.00

**Table 2:** Characteristics of tested set of samples.

	Ciders		Other Alcoholic Beverages	Juice	
	Home-Made	Commercial		Home-Made	Commercial
n	6	25	40	1	1
**Total**	**31**		**40**	**2**	

**Table 3:** ICP-MS (Thermo Electron Corporation, X SERIES, UK) and ICP-OES (Thermo Scientific, ICAP 7000 series, MA, USA) parameters and measurement conditions.

Parameter and Accessories	ICP MS	ICP OES
Number of replicates	3	3
Carrier gas	Argon	Argon
Plasma gas flow rate [L·min^−1^]	12.9	12
Auxiliary gas flow rate [L·min^−1^]	0.84	0.5
Nebulization gas flow rate [L·min^−1^]	0.92	0.5
Torch	Quartz	Quartz
Nebulizer	Concentric quartz	Concentric quartz
Generator power [W]	1380	1150
Internal standard	In	In

**Table 4:** Contents of selected elements in the measured cider samples and low-percentage alcoholic beverages (n = 73) [µg/L].

Elements	Concentration Unit	Mean	Median	Min	Max	Std. Dev.
Ag	µg/L	0.927	0.848	<LOD	2.569	0.688
Al	µg/L	1249	1221	417.1	1953	319.2
B	µg/L	1201	1162	477.3	2033	380.7
Ba	µg/L	164.7	144.7	71.58	465.2	93.29
Bi	µg/L	0.398	0.194	<LOD	3.526	0.589
Ca	mg/L	58.57	45.53	25.58	159.6	35.01
Cd	µg/L	0.707	0.349	0.046	3.074	0.783
Co	µg/L	2.601	0.943	0.032	13.42	3.226
Cr	µg/L	14.30	10.54	1.636	74.91	11.07
Cu	µg/L	60.33	34.01	0.779	490.0	90.33
Fe	mg/L	1.420	0.621	<LOD	16.59	2.521
Hg	µg/L	<LOD	<LOD	<LOD	<LOD	<LOD
K	mg/L	418.2	310.9	24.86	1053	283.49
Li	µg/L	1.303	1.060	0.089	9.078	1.121
Mg	mg/L	48.87	47.29	17.30	114.8	19.96
Mn	µg/L	222.4	135.7	31.26	649.5	180.1
Na	mg/L	83.50	80.50	11.46	334.3	54.66
Ni	µg/L	50.99	15.21	1.581	199.2	63.68
Pb	µg/L	5.914	2.114	<LOD	18.72	9.843
Sr	µg/L	109.7	19.15	3.266	1217	269.6
Ti	µg/L	5.328	3.124	<LOD	30.42	6.698
Tl	µg/L	0.058	0.045	<LOD	0.509	0.070
Zn	µg/L	126.5	18.24	2.521	606.0	169.5

LOD—limit of detection.

**Table 5:** Comparison ranges of selected elements in ciders [6,19] and beers [13,28] indicated in other papers with results obtained in this study.

	This Study				García-Ruiz et al., 2007 [19] (Ciders)		Cristea et al., 2019 [6] (Ciders)		Alexa et al., 2018 [13] (Beers)		Sakellari et al., 2017 [28] (Beers)	
	Others		Ciders									
Element	Min	Max	Min	Max	Min	Max	Min	Max	Min	Max	Min	Max
Ag	0.046	2.569	<LOD	1.852	−	−	−	−	−	−	−	−
Al	854.4	1905	417.1	1954	6.4	1000	39.34	1289	<LOD	92.8	−	−
B	477.3	2033	486.7	1826	210	4200	−	−	−	−	−	−
Ba	71.58	211.2	76.13	460.1	4.9	330	−	−	9.95	39.9	11	56
Bi	<LOD	3.526	0.033	1.386	<LOD	0.49	−	−	−	−	−	−
Cd	0.046	0.422	0.097	3.074	<LOD	3.4	−	−	<LOD	<LOD	<LOD	1
Co	0.032	1.408	0.275	13.42	<LOD	3.9	−	−	0.169	0.481	<LOD	1.1
Cr	2.258	74.91	1.636	34.86	<LOD	18	<LOD	247	0.919	9.36	1.7	48
Cu	4.937	95.38	5.080	490.0	<LOD	180	<LOD	240.5	27.3	109	<LOD	84
Hg	<LOD	<LOD	<LOD	<LOD	−	−	−	−	−	−	−	−
Li	0.274	2.146	0.090	9.078	<LOD	8.7	−	−	−	−	−	−
Mn	38.29	269.0	31.26	649.5	18	470	29.68	475.7	41	260	44	377
Ni	3.161	111.1	1.581	199.2	<LOD	110	<LOD	523.7	<LOD	11.2	3.1	40
Pb	<LOD	16.93	<LOD	17.05	0.44	32	<LOD	14.86	<LOD	6.01	0.39	11
Sr	4.881	29.57	3.266	1217	4.4	470	−	−	<LOD	212	58	292
Ti	<LOD	24.02	<LOD	30.42	0.62	33	−	−	−	−	−	−
Tl	<LOD	0.078	0.017	0.509	<LOD	0.36	−	−	−	−	−	−
Zn	2.521	92.24	7.216	533.2	8.7	560	6.7	407.9	23.9	98.1	<LOD	105
*Ca	31.09	140.4	25.58	159.6	14	180	0.15	68.89	−	−		
*Fe	<LOD	1.298	0.163	16.59	0.034	2	<LOD	7.68	<LOD	<LOD	0.058	0.838
*K	24.86	489.5	237.3	1050	74	1300	67.89	555.9	−	−	−	−
*Mg	21.52	114.79	17.30	79.38	16	69	5.25	50.29	−	−	−	−
*Na	17.12	334.3	11.46	175.4	3.4	190	5.74	170.2	−	−	−	−

* contents are given in mg/L. LOD—limit of detection.

**Table 6:** Contents of selected elements (with statistically significant differences) in the measured home-made cider (n = 6) and commercial cider samples (n = 25) [µg/L].

Element	Type	n	Mean	Median	Min	Max	Std. Dev
**Ag**	Commercial	25	0.827	0.802	< LOD	1.852	0.579
	Home-made	6	0.183	0.135	0.073	0.472	0.145
**Al**	Commercial	25	1316	1273	994.6	1954	264.5
	Home-made	6	622.1	597.6	417.1	916.6	174.1
**B**	Commercial	25	1259	1186	851.0	1825	299.8
	Home-made	6	689.0	645.1	486.7	935.8	181.9
**Bi**	Commercial	25	0.319	0.244	0.033	1.386	0.291
	Home-made	6	0.741	0.695	0.415	1.256	0.328
**Co**	Commercial	25	4.317	3.963	0.590	13.42	3.172
	Home-made	6	7.592	7.015	4.382	11.53	3.270
**Cr**	Commercial	25	18.42	23.16	4.934	34.86	8.522
	Home-made	6	25.08	24.71	24.17	27.33	1.154
**Cu**	Commercial	25	48.46	44.00	5.080	164.9	35.20
	Home-made	6	254.7	236.5	97.00	490.0	170.3
*Fe	Commercial	25	2.615	1.993	0.218	16.60	3.466
	Home-made	6	4.121	2.840	2.411	9.090	2.589
*K	Commercial	25	563.1	575.7	207.1	797.8	161.4
	Home-made	6	991.2	1001	918.7	1050	54.91
**Li**	Commercial	25	1.487	0.880	0.503	9.078	1.762
	Home-made	6	1.855	1.947	1.390	2.358	0.362
*Mg	Commercial	25	37.88	36.69	17.30	65.02	12.05
	Home-made	6	60.05	59.15	44.31	79.38	16.77
*Na	Commercial	25	106.6	112.4	24.15	175.4	37.73
	Home-made	6	22.63	25.04	11.46	31.48	8.507
**Ni**	Commercial	25	75.96	59.60	6.093	152.5	57.14
	Home-made	6	188.8	188.4	180.2	199.2	6.794
**Ti**	Commercial	25	5.955	4.800	< LOD	30.42	6.627
	Home-made	6	11.55	11.35	5.200	18.80	5.773
**Zn**	Commercial	25	205.3	256.0	7.216	533.2	152.4
	Home-made	6	395.7	407.0	277.0	494.0	74.15

* contents are given in mg/L. LOD—limit of detection.

## Data Availability

Not applicable.

## Supplementary Materials

The following are available online, Figures S1, S2 (from A to O), S3 (from A to J) and S4; Tables S1–S4.

## Sample Availability

Samples of the compounds are available from the authors.
