# Supplementary material for: Elemental Characterization of Ciders and Other Low-Percentage Alcoholic Beverages Available on the Polish Market
Source: Molecules. 2021 Apr 10;26(8):2186. doi: 10.3390/molecules26082186 (PMC8070395; doi:10.3390/molecules26082186)
Supplement: Supplementary file 1 [file molecules-26-02186-s001.pdf]

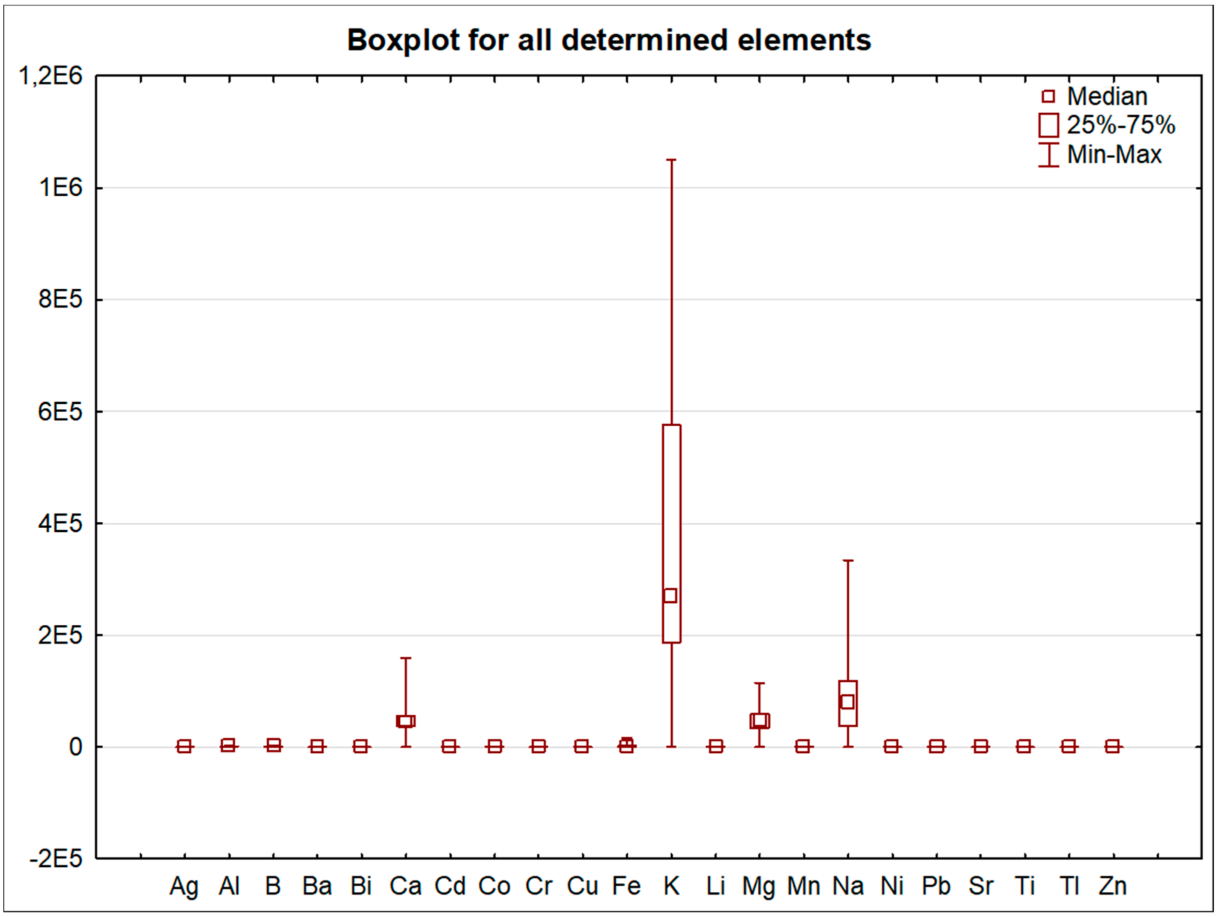

Figure S1. Boxplot for content of all determined elements in 73 samples of ciders, apple juice and other low-percentage alcohol beverages [μg/L].

Figure S2. Boxplot for contents of selected elements (with statistically significant differences) in the measured home-made cider (n=6) and commercial ciders samples (n=25) A-Ag; B-Al, C-B; D-Bi; E-Co; F-Cr; G-Cu; H-Fe; I-K; J-Li; K-Mg; L-Na; M-Ni; N-Ti; O-Zn.

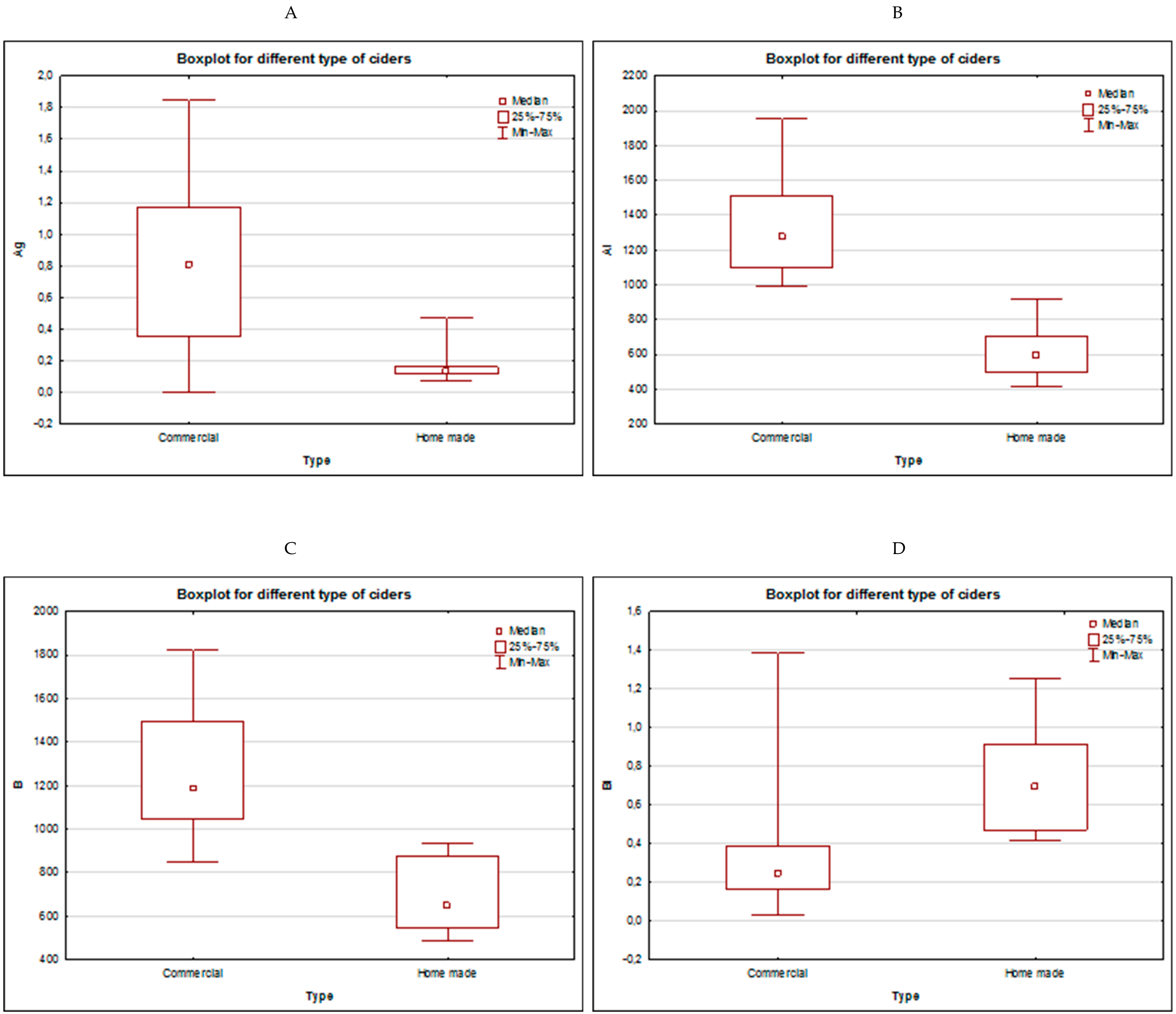

E

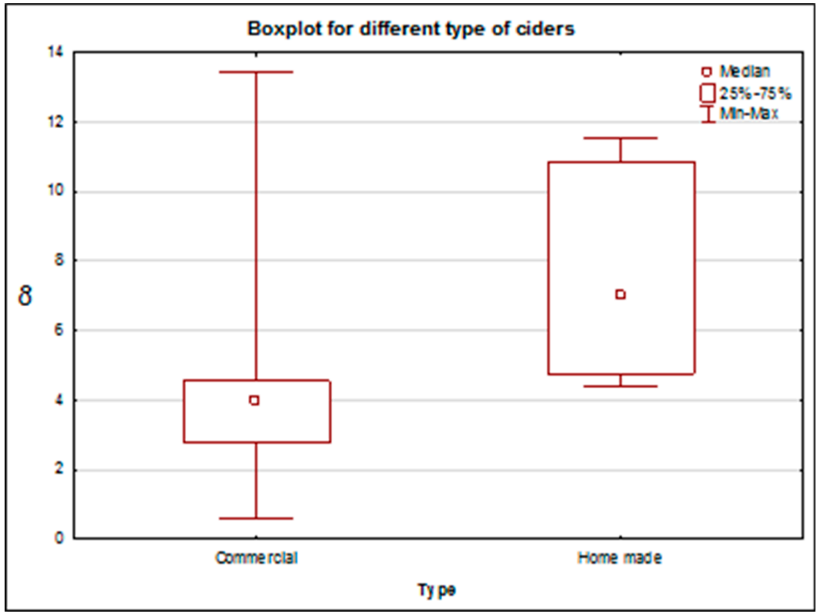

Boxplot for different type of ciders
Median
25%-75%
Min-Max
Commercial
Home made
Type

F

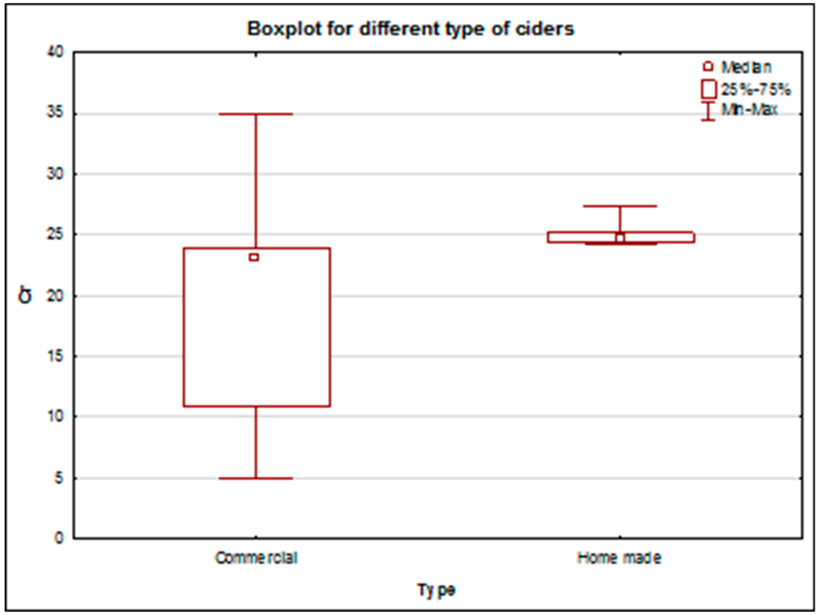

Boxplot for different type of ciders
Median
25%-75%
Min-Max
Commercial
Home made
Type

G

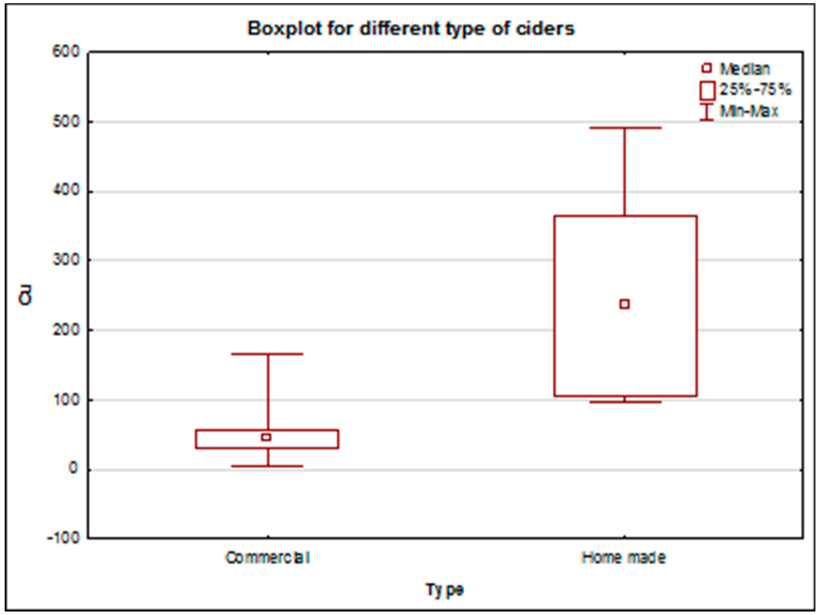

Boxplot for different type of ciders
Median
25%-75%
Min-Max
Commercial
Home made
Type

H

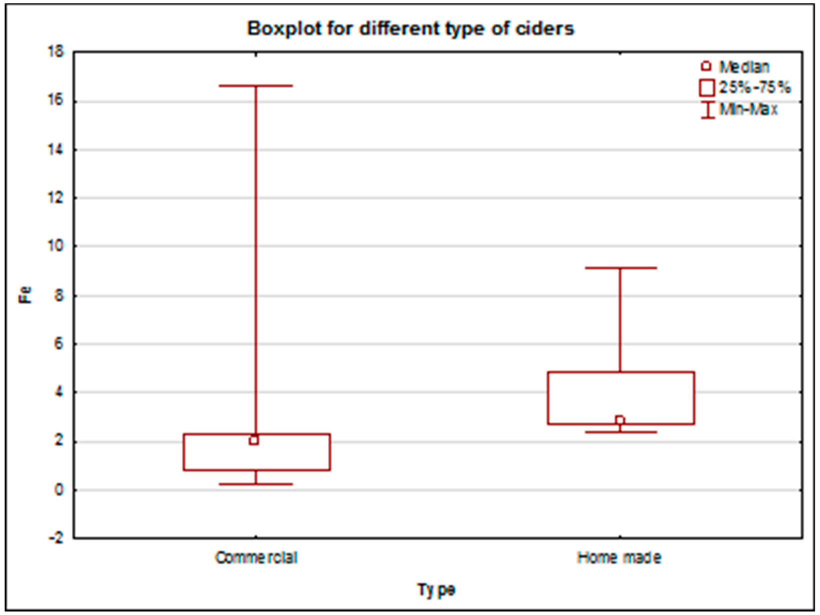

Boxplot for different type of ciders
Median
25%-75%
Min-Max
Fe
Commercial
Home made
Type

I

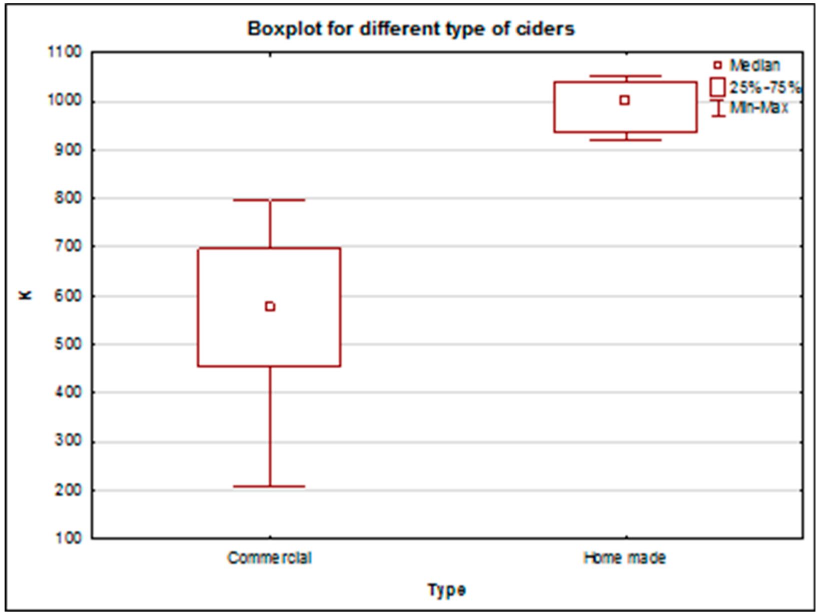
Boxplot for different type of ciders
Median
25%-75%
Min-Max
K
1100
1000
900
800
700
600
500
400
300
200
100
Commercial
Home made
Type

J

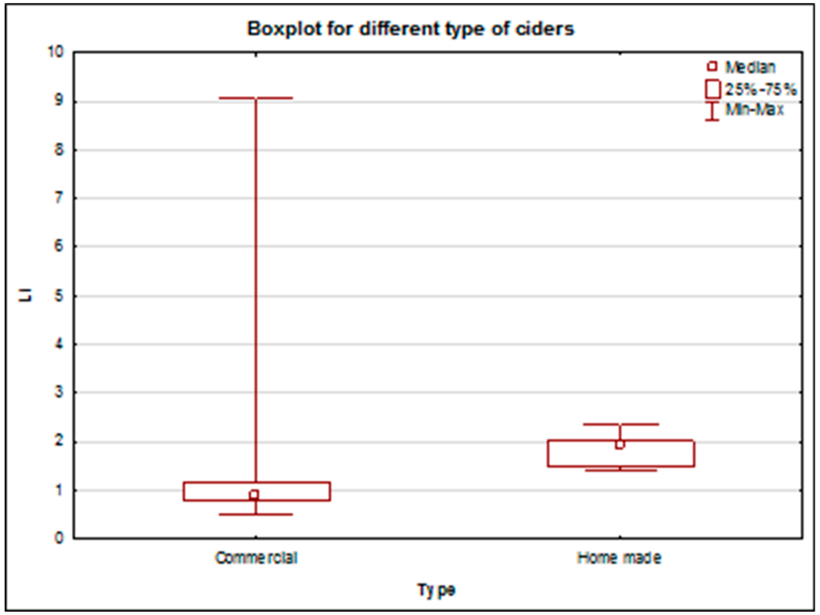
Boxplot for different type of ciders
Median
25%-75%
Min-Max
Li
10
9
8
7
6
5
4
3
2
1
0
Commercial
Home made
Type

K

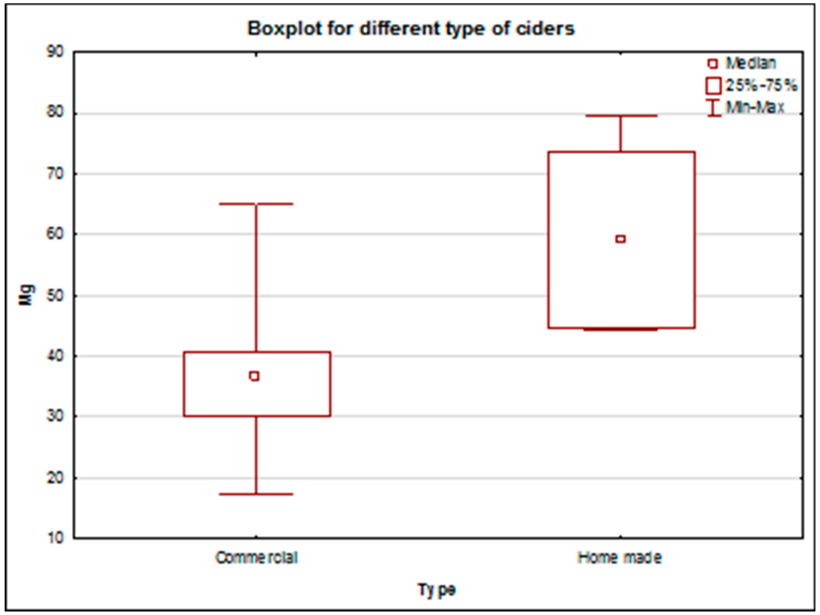
Boxplot for different type of ciders
Median
25%-75%
Min-Max
Mg
90
80
70
60
50
40
30
20
10
Commercial
Home made
Type

L

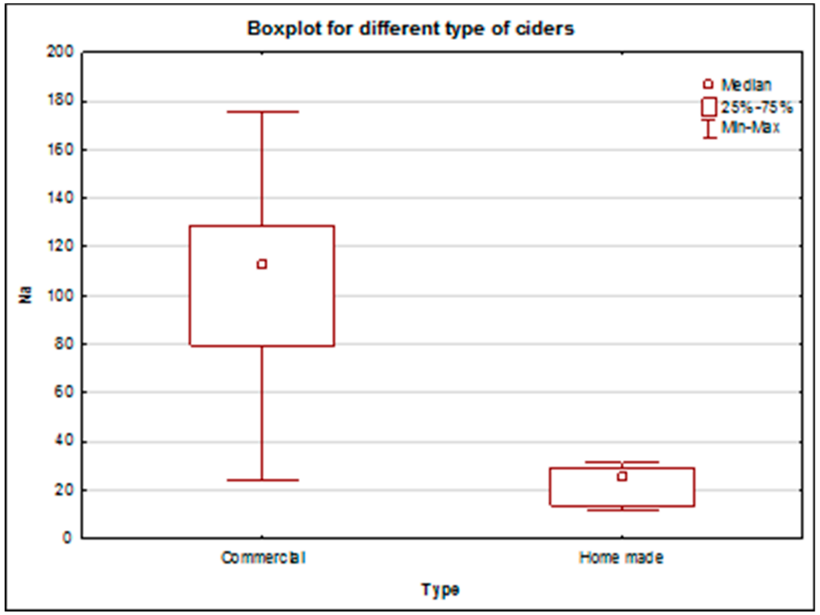
Boxplot for different type of ciders
Median
25%-75%
Min-Max
Na
200
180
160
140
120
100
80
60
40
20
0
Commercial
Home made
Type

M

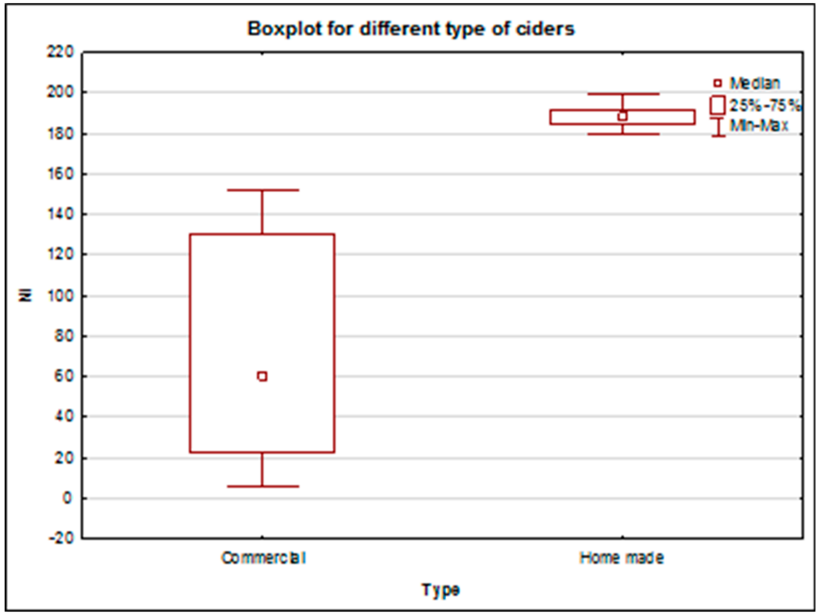
Boxplot for different type of ciders
Median
25%-75%
Min-Max
Ni
Commercial
Home made
Type

N

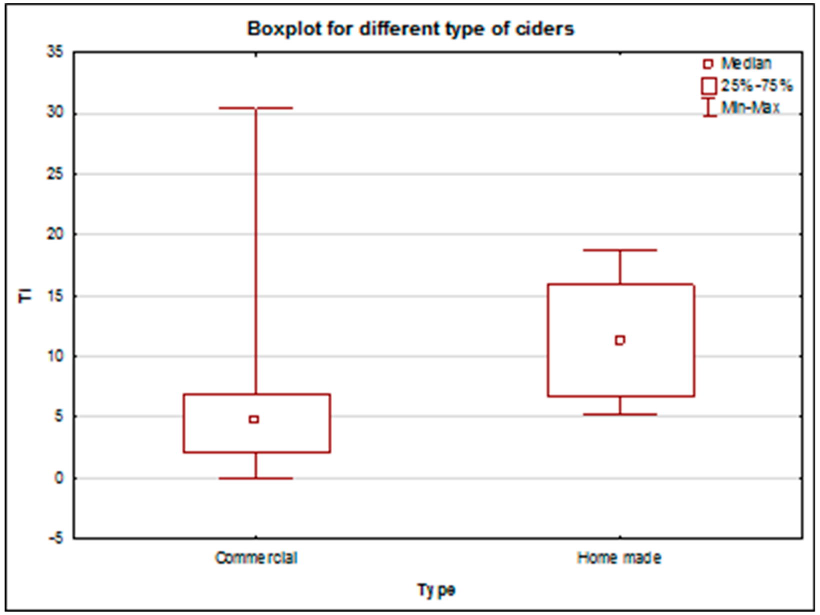
Boxplot for different type of ciders
Median
25%-75%
Min-Max
Tl
Commercial
Home made
Type

O

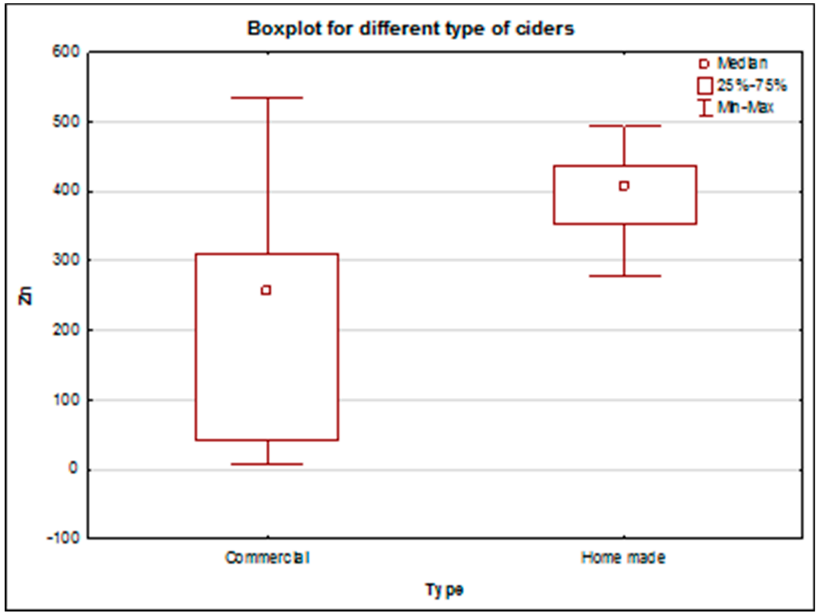
Boxplot for different type of ciders
Median
25%-75%
Min-Max
Zn
Commercial
Home made
Type

Table S1. Contents of selected elements (with statistically significant differences) in the measured home-made cider (n=6).

Element	Code	n	Mean	Median	Min	Max	Std. Dev
Ba	CR	4	445.5	446.9	422.8	465.2	20.49
	CL	4	149.6	143.1	111.0	201.1	39.25
Ca	CR	4	123.7	123.3	120.4	127.9	3.290
	CL	4	38.44	38.03	37.71	40.00	1.050
Co	CR	4	9.830	9.960	7.870	11.53	1.670
	CL	4	4.660	4.640	4.380	4.960	0.260
Cu	CR	4	109.5	109.5	97.00	122.0	11.15
	CL	4	405.5	387.0	358.0	490.0	60.93
K	CR	4	932.1	927.4	893.1	980.3	36.69
	CL	4	1041	1044	1023	1053	13.72
Li	CR	4	1.980	2.000	1.550	2.360	0.330
	CL	4	1.500	1.430	1.220	1.910	0.290
Mg	CR	4	74.59	73.17	72.64	79.38	3.220
	CL	4	45.60	45.18	44.31	47.74	1.530
Mn	CR	4	565.7	573.0	537.6	579.1	19.31
	CL	4	353.5	350.3	341.3	372.2	13.32
Na	CR	4	28.97	28.81	26.79	31.48	1.940
	CL	4	15.85	15.17	11.46	21.61	4.400
Sr	CR	4	1190	1199	1147	1217	30.63
	CL	4	49.34	50.14	45.32	51.76	2.990

Figure S3. Boxplot for contents of selected elements (with statistically significant differences) in the measured home-made cider (n=6)
A-Ba; B-Ca; C-Co; D-Cu; E-K; F-Li; G-Mg; H-Mn; I-Na; J-Sr.

A

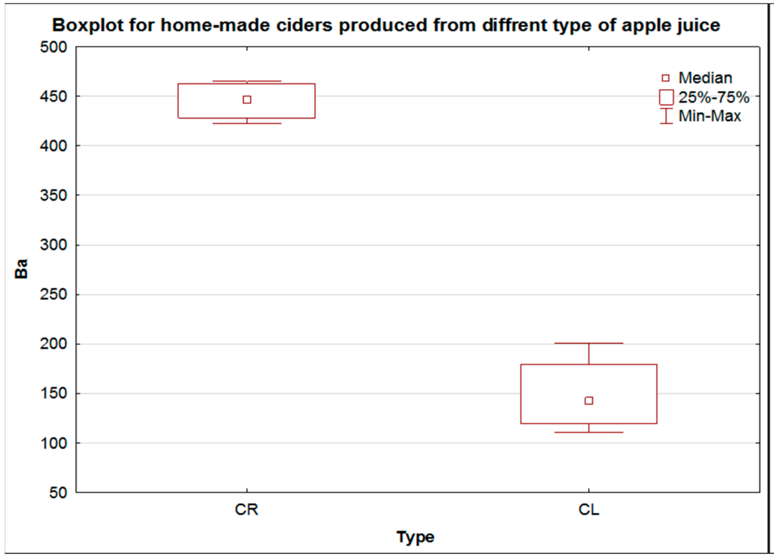

B

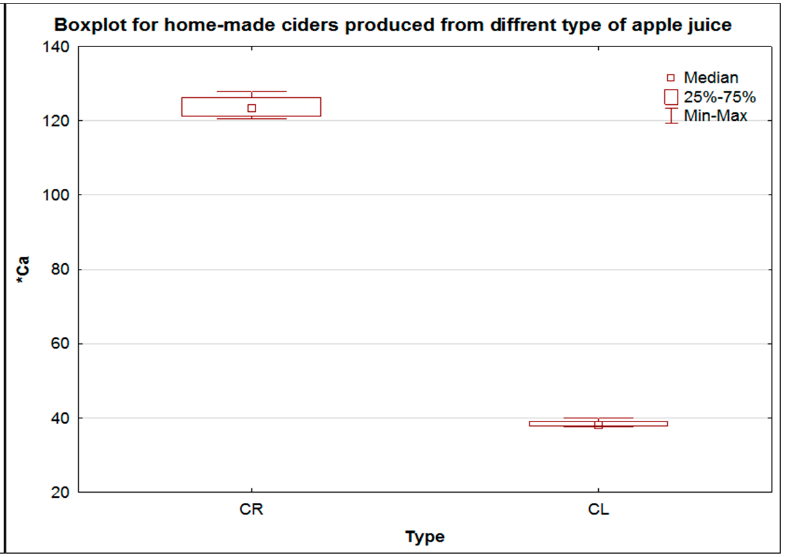

C

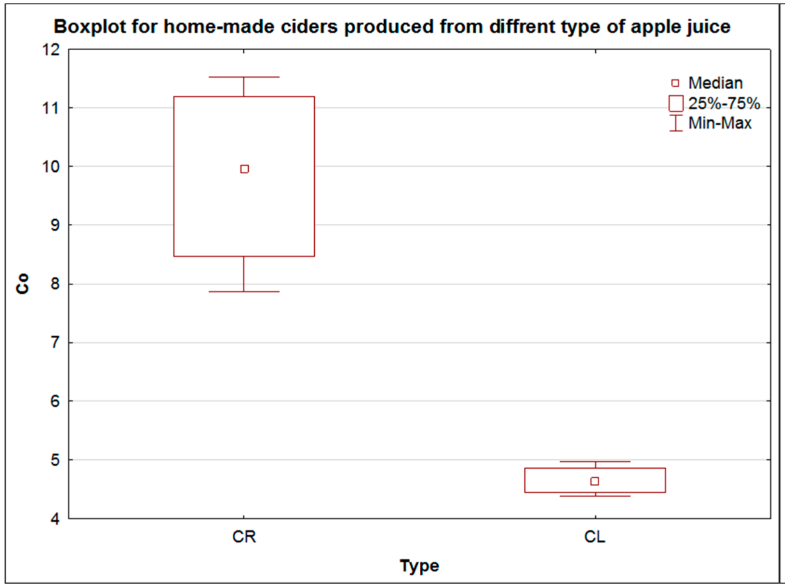

D

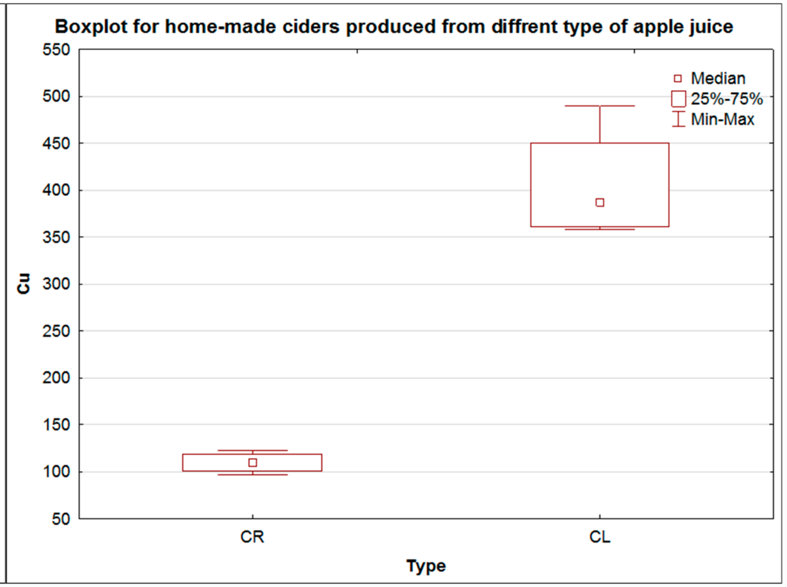

E

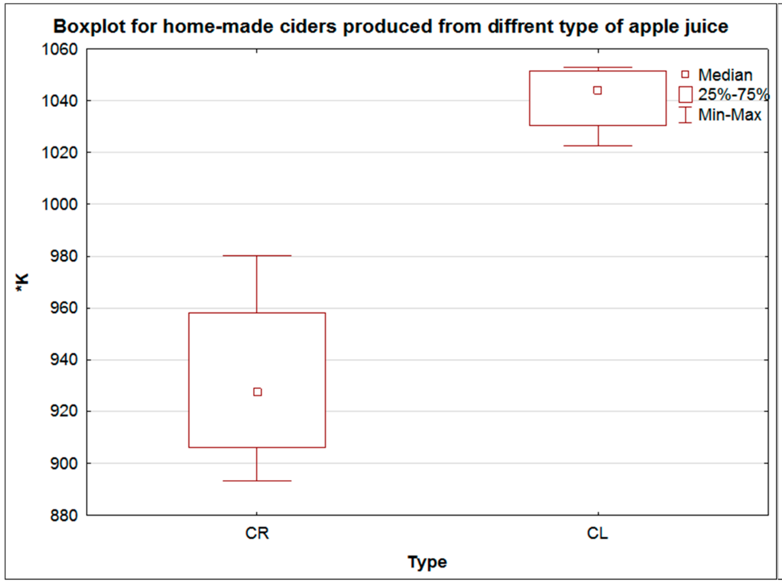
Boxplot for home-made ciders produced from diffrent type of apple juice
1060
1040
1020
1000
980
960
940
920
900
880
*K
Median
25%-75%
Min-Max
CR
CL
Type

F

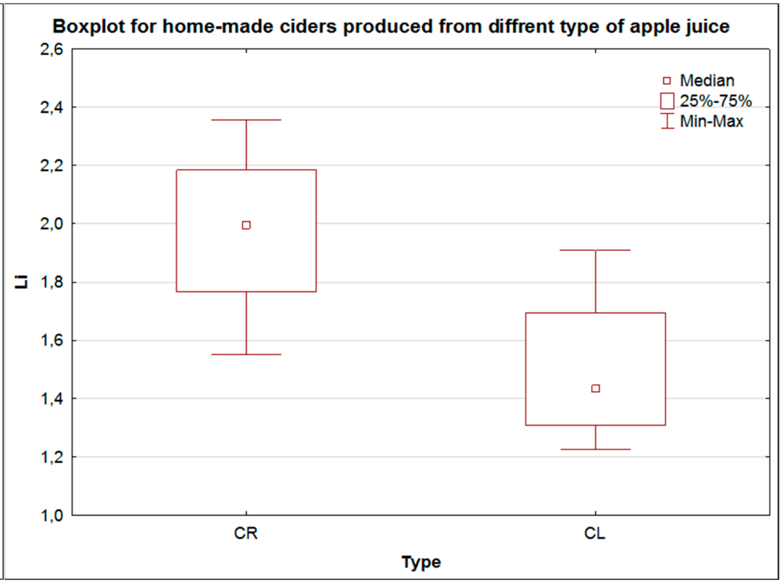
Boxplot for home-made ciders produced from diffrent type of apple juice
2,6
2,4
2,2
2,0
1,8
1,6
1,4
1,2
1,0
Li
Median
25%-75%
Min-Max
CR
CL
Type

G

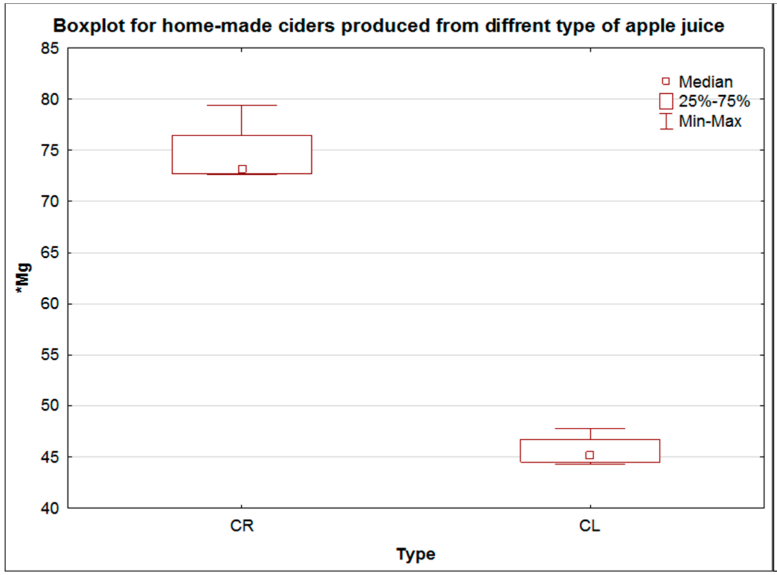
Boxplot for home-made ciders produced from diffrent type of apple juice
85
80
75
70
65
60
55
50
45
40
*Mg
Median
25%-75%
Min-Max
CR
CL
Type

H

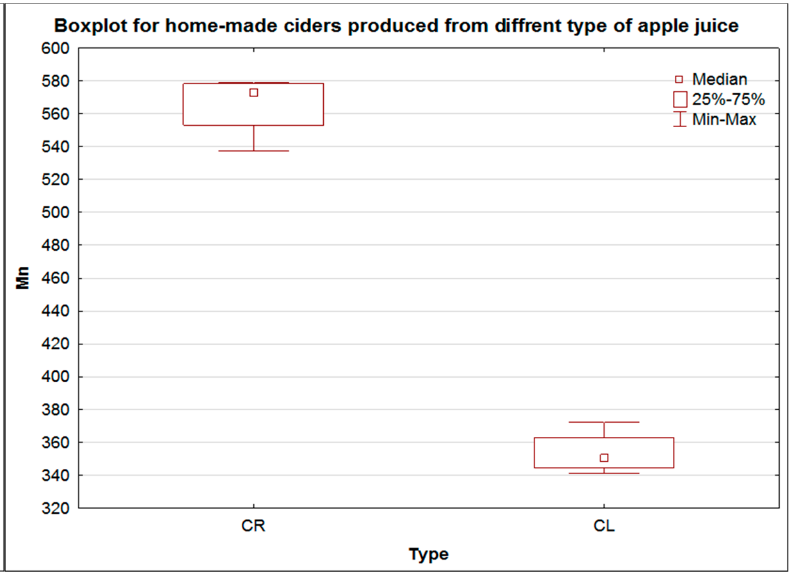
Boxplot for home-made ciders produced from diffrent type of apple juice
600
580
560
540
520
500
480
460
440
420
400
380
360
340
320
Mn
Median
25%-75%
Min-Max
CR
CL
Type

I

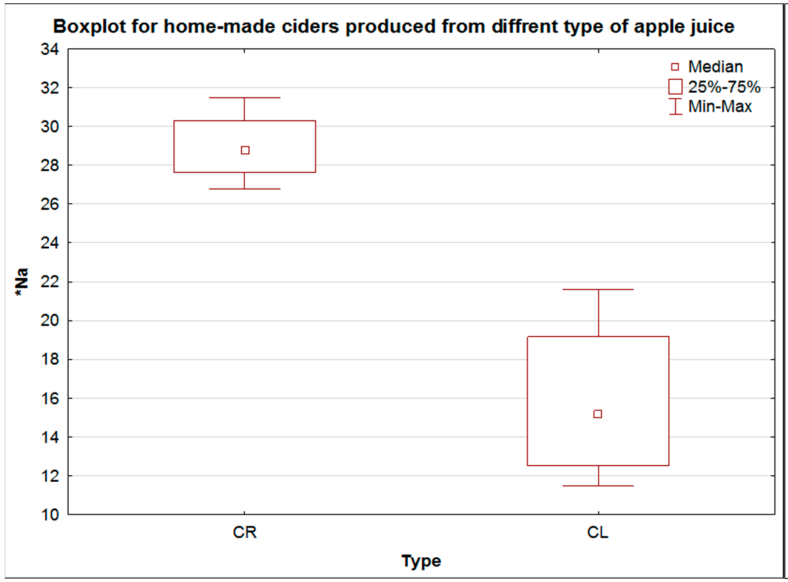

J

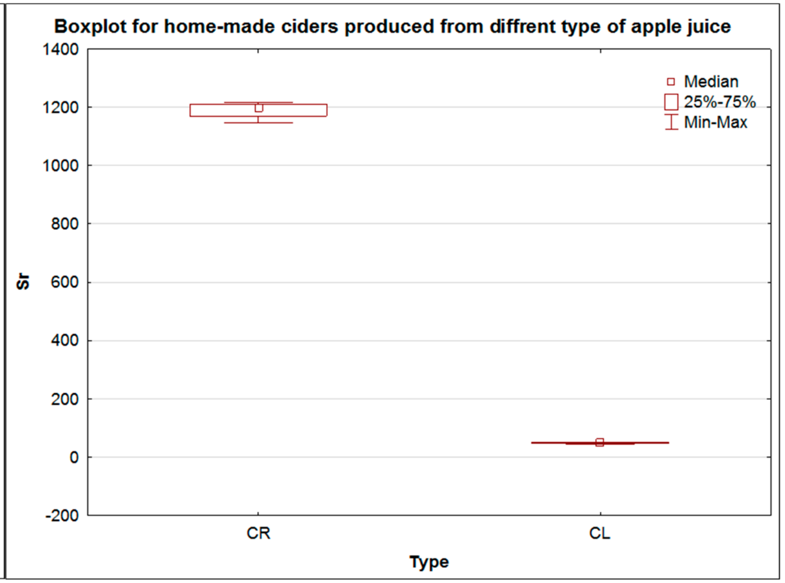

Table S2. Basic statistics of Cu, Mn and Na content for ciders samples with division according to type of package (n = 12) [μg/L].

Element	Package	n	Mean	Median	Min	Max	Std. Dev.
Cu	Bottle (Green)	6	71.98	64.00	49.00	132.9	31.15
	Can	6	48.80	49.00	40.78	56.00	5.768
Mn	Bottle (Green)	6	431.7	424.7	319.0	577.3	110.9
	Can	6	571.2	576.4	478.5	649.5	54.75
Na	Bottle (Green)	6	119.3	119.8	106.1	130.8	10.55
	Can	6	98.06	105.3	71.40	118.5	18.43

Table S3. Basic statistical parameters of the pH results of all tested alcohol.

Type	n	Mean	Median	Min	Max	Std. dev.
Other	39	3.122	3.027	2.617	4.030	0.302
Cider	32	3.323	3.290	2.980	3.767	0.176
Commercial	25	3.295	3.260	3.000	3.767	0.162
Home made	6	3.497	3.510	3.440	3.560	0.048
CR	3	3.460	3.440	3.440	3.500	0.035
CL	3	3.533	3.530	3.510	3.560	0.025
JR	1	3.400	3.400	3.400	3.400	-
JL	1	3.530	3.530	3.530	3.530	-

Figure S4. Projection of the cases on the factor-plane in 79 samples investigated in this study according to their producer code and for water samples.

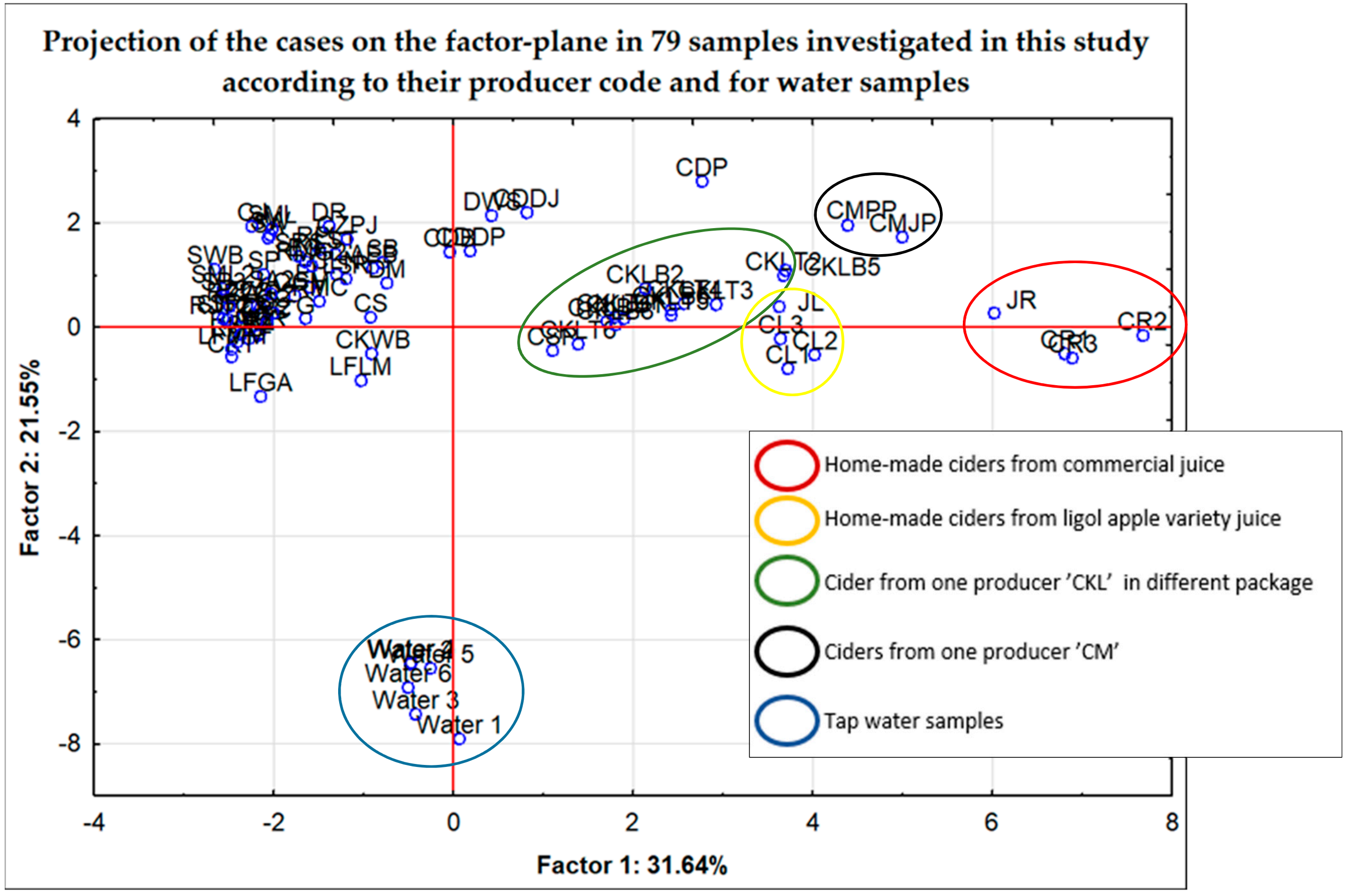

Table S4. The characterization of the alcohol samples [µg/L]; *[mg/L].

No.	Code	Package	%	Acidity regulator	Type	Ag	Al	B	Ba	Bi	*Ca	Cd	Co	Cr	Cu	*Fe	Hg	*K	Li	*Mg	Mn	*Na	Ni	Pb	Sr	Ti	Tl	Zn
1	SRS	Bottle (White)	4.5	Citric acid	Other	1.665	1304	1519	126.9	0.263	55.61	0.155	0.352	8.246	32.53	0.176	>LOD	237.1	1.329	55.40	108.0	90.44	9.427	<LOD	20.93	19.48	0.026	8.238
2	SWB	Bottle (White)	0	Malic acid. Citric acid	Other	2.309	1547	1779	157.1	0.179	38.29	0.333	0.352	6.564	12.78	0.407	<LOD	109.2	1.485	24.69	65.21	85.18	11.82	0.198	15.21	<LOD	0.057	12.35
3	SP	Bottle (White)	0	Citric acid	Other	1.892	1542	1771	170.1	0.139	59.72	0.334	0.776	7.620	29.90	0.037	<LOD	175.5	1.687	31.61	75.02	69.17	13.12	2.700	20.66	<LOD	0.055	10.48
4	SA	Bottle (White)	4.5	Citric acid. Malic acid. Ascorbic acid	Other	2.472	1521	1691	148.9	0.154	43.15	0.295	0.532	10.54	28.08	0.715	<LOD	217.5	1.888	59.99	135.7	86.62	10.79	0.532	15.73	6.901	0.032	5.672
5	SML	Bottle (White)	4.5	Citric acid	Other	2.063	1810	2033	211.2	0.194	53.26	0.362	0.395	8.292	34.01	0.348	<LOD	230.4	1.829	52.82	116.6	87.67	9.010	2.561	19.86	<LOD	0.045	11.83
6	SW	Bottle (White)	4.5	Citric acid	Other	2.177	1664	1869	202.0	0.075	47.36	0.422	0.388	8.444	30.90	0.630	<LOD	233.2	1.719	54.06	111.4	106.7	9.710	1.327	19.15	<LOD	0.067	8.810
7	RPL	Bottle (White)	4	Citric acid	Other	1.892	1558	1788	198.7	0.144	51.96	0.363	0.446	8.921	46.89	1.296	<LOD	199.2	1.263	44.06	88.61	103.7	9.572	9.545	18.37	3.146	0.047	17.01
8	EL	Can	2.5	Citric acid	Other	1.554	1124	1367	167.0	0.132	56.02	0.244	0.501	6.435	46.82	0.063	<LOD	324.2	1.873	63.36	178.6	72.97	16.64	<LOD	26.47	5.076	0.034	5.847
9	EG	Can	2.5	Citric acid. Magnesium carbonate	Other	1.469	1098	1289	174.1	0.092	41.69	0.249	0.210	8.764	35.66	0.012	<LOD	257.2	1.362	96.03	137.8	105.1	15.78	<LOD	19.15	6.841	0.054	14.05
10	CJ	Bottle (White)	6	Citric acid. Sodium citrate	Other	1.207	1242	1489	133.3	0.077	36.47	0.396	0.185	9.711	39.50	0.113	<LOD	270.6	0.983	57.86	95.08	334.3	7.047	<LOD	11.71	5.665	0.049	8.680
11	AEB	Can	6	Citric acid	Other	0.452	1239	1476	150.9	0.182	37.34	0.138	0.308	10.78	52.65	0.394	<LOD	473.3	1.587	100.4	196.8	67.92	25.55	6.603	14.38	<LOD	0.056	13.62
12	DR	Bottle (White)	6	Citric acid	Other	0.752	1577	1768	169.9	0.136	114.3	0.298	0.687	10.59	52.02	0.044	<LOD	352.1	1.004	80.08	126.6	145.6	12.57	0.790	20.62	0.623	0.039	3.195
13	ŁG	Bottle (Green)	0	Citric acid. Magnesium carbonate	Other	1.113	931	1077	140.9	0.267	35.45	0.153	0.213	4.573	41.289	<LOD	<LOD	182.3	1.181	70.25	89.28	125.4	14.01	<LOD	14.58	0.620	0.039	7.031
14	ŁOCM	Bottle (Green)	0	Citric acid	Other	1.583	1176	1353	169.9	0.271	38.76	0.318	0.325	13.75	43.67	<LOD	<LOD	196.8	1.669	46.65	121.1	80.49	20.52	1.712	17.69	<LOD	0.063	6.663
15	PWRZ	Brown bottle	0	Citric acid	Other	2.351	1205	1422	196.1	0.220	35.15	0.303	0.417	6.938	23.08	<LOD	<LOD	123.1	2.146	35.83	81.92	32.15	10.02	1.691	29.30	<LOD	0.078	5.025
16	LSR	Can	5	Citric acid	Other	1.147	1033	1231	141.1	0.141	40.98	0.252	1.209	8.294	44.73	1.230	<LOD	421.2	1.808	93.28	269.0	82.94	13.53	0.258	19.01	<LOD	0.048	16.16
17	LST	Can	5	Citric acid	Other	1.359	1120	1080	144.7	0.114	60.52	0.375	0.330	11.33	56.19	<LOD	<LOD	489.6	1.888	114.8	150.4	147.2	14.74	2.222	29.57	3.090	0.014	12.38
18	CDDP	Bottle (White)	4.5	-	Cider	1.689	1581	1653	301.9	0.256	67.26	0.713	2.604	7.134	41.46	0.630	<LOD	537.7	2.025	39.51	282.1	138.6	31.19	15.23	20.45	<LOD	0.070	43.07
19	CDDJ	Bottle (White)	4.5	-	Cider	1.606	1739	1826	395.2	0.166	139.6	0.499	2.757	13.01	33.21	1.842	<LOD	473.8	2.051	49.05	265.5	129.7	22.38	9.558	35.61	6.161	0.079	9.198
20	CKLB1	Bottle (Green)	4.5	-	Cider	0.390	1016	1144	226.5	0.244	38.54	2.500	3.963	29.26	132.9	1.981	<LOD	534.8	1.044	32.82	319.0	128.3	115.2	7.882	111.4	6.585	0.116	240.1
21	CZPJ	Bottle (White)	4.5	-	Cider	1.852	1709	1791	266.7	0.194	31.82	0.374	0.943	7.213	5.080	0.718	<LOD	434.2	0.818	27.54	264.9	112.4	8.422	7.754	12.59	<LOD	0.061	8.327
22	CSP	Bottle (White)	4.5	-	Cider	1.576	1485	1504	278.5	0.222	70.21	1.666	3.006	12.13	164.8	0.621	<LOD	360.7	9.078	36.45	189.6	118.9	24.07	14.01	262.8	14.29	0.051	42.15

23	CMJP	Bottle (Brown)	4.5	-	Cider	1.656	1530	1551	298.9	0.402	155.1	0.709	12.36	25.15	30.95	16.59	<LOD	527.8	3.463	61.06	431.4	40.77	48.22	12.28	287.8	3.124	0.217	433.7
24	CMPP	Bottle (Brown)	4.5	-	Cider	1.628	1395	1220	224.7	0.475	159.6	1.064	13.42	21.65	20.38	9.938	<LOD	586.8	3.282	65.02	321.4	75.23	59.99	7.095	234.3	16.92	0.165	533.2
25	CS	Bottle (Green)	4.5	-	Cider	1.105	1273	1186	87.60	0.145	75.41	0.262	5.816	12.64	12.67	0.414	<LOD	797.8	0.794	17.30	61.76	24.15	14.92	<LOD	9.075	<LOD	0.041	277.2
26	CDB	Bottle (White)	4.5	-	Cider	1.017	1512	1497	208.9	0.097	155.7	0.494	3.105	7.725	29.79	0.957	<LOD	586.0	1.060	60.23	325.5	57.11	28.19	1.221	24.62	2.485	0.027	37.61
27	CDP	Bottle (White)	4.5	-	Cider	1.174	1954	1474	199.0	0.196	145.5	0.650	9.053	34.86	57.88	4.221	<LOD	452.8	1.724	43.44	331.9	175.4	29.51	14.97	36.20	30.42	0.070	347.6
28	CLN	Bottle (White)	4.5	-	Cider	0.982	1581	1744	122.2	0.117	36.05	0.399	1.483	5.705	16.20	1.267	<LOD	498.1	0.503	33.58	205.0	44.69	12.60	8.331	6.986	<LOD	0.036	42.31
29	ŁRC	Bottle (Green)	2	-	Other	0.803	996.0	851.0	90.80	0.104	37.57	0.153	0.978	4.934	32.31	0.218	<LOD	207.1	0.892	47.29	71.70	128.9	17.77	<LOD	14.60	8.099	0.022	44.42
30	AMR	Can	2	Citric acid	Other	0.885	1169	1259	108.9	0.095	64.08	0.184	1.408	4.451	24.85	0.560	<LOD	155.2	0.823	36.19	38.29	20.83	13.22	<LOD	15.95	<LOD	0.042	68.66
31	RMG	Bottle (White)	4.5	Citric acid	Other	0.961	1054	909.4	82.00	0.023	40.79	0.085	0.373	10.27	22.13	<LOD	<LOD	176.4	0.665	48.10	88.84	17.12	8.368	<LOD	10.63	<LOD	<LOD	7.197
32	SB	Can	4.5	Citric acid	Other	1.091	1079	892.9	80.40	0.022	46.52	0.152	0.793	74.91	17.45	1.298	<LOD	178.8	1.206	53.78	102.1	159.0	47.58	<LOD	11.44	<LOD	0.011	9.604
33	G	Bottle (White)	4.6	Citric acid. Ascorbic acid	Other	0.812	1063	855.6	77.90	0.042	140.4	0.162	0.482	8.210	5.927	0.019	<LOD	228.4	0.749	64.00	77.24	44.39	13.65	<LOD	21.72	7.544	<LOD	8.664
34	CC	Bottle (White)	4.5	-	Cider	0.802	1199	961.6	96.30	0.036	55.83	0.223	0.955	10.91	26.94	0.702	<LOD	310.9	0.596	22.57	135.8	155.7	7.725	<LOD	14.13	0.627	0.019	13.44
35	CC2	Bottle (White)	4.5	-	Cider	0.646	1128	951.1	101.7	0.033	55.64	0.158	0.590	8.407	15.18	0.818	<LOD	325.7	0.519	20.90	119.5	158.8	6.093	<LOD	12.83	1.258	0.017	7.216
36	SB2	Can	4.5	Citric acid	Other	0.902	1064	919.2	90.30	0.016	49.93	0.108	0.405	5.109	13.45	1.270	<LOD	186.9	1.114	53.87	89.57	36.15	8.010	<LOD	12.27	<LOD	<LOD	5.383
37	SA2	Can	4.5	Citric acid. Malic acid. Ascorbic acid	Other	0.964	1431	1220	98.30	0.033	45.53	0.139	0.216	5.469	14.57	0.188	<LOD	188.4	1.016	53.03	79.14	33.28	5.286	<LOD	11.44	18.84	<LOD	5.927
38	SML2	Can	4.5	Citric acid	Other	0.933	1459	1281	106.5	0.033	51.00	0.142	0.687	4.757	21.10	0.044	<LOD	193.5	1.376	52.92	68.69	140.0	6.066	<LOD	13.17	<LOD	0.011	40.97
39	SB3	Bottle (White)	4.5	Citric acid	Other	0.891	1439	1250	99.70	<LOD	51.73	0.303	0.538	4.904	18.26	0.243	<LOD	24.86	1.180	62.43	93.33	92.23	6.125	<LOD	12.11	3.115	<LOD	8.494
40	SPFO	Bottle (White)	4.5	Citric acid. Ascorbic acid	Other	0.759	1221	1060	89.40	0.057	31.09	0.285	1.341	5.502	24.17	0.395	<LOD	196.9	1.112	59.75	91.28	85.16	8.578	<LOD	9.803	<LOD	0.011	11.78
41	RJTC	Can	4.5	Citric acid	Other	0.496	1223	1050	87.00	0.055	39.31	0.138	0.535	5.497	20.73	<LOD	<LOD	178.3	0.578	46.73	71.31	34.37	5.381	1.037	9.370	3.114	<LOD	15.36
42	RŻ	Can	4.5	Citric acid	Other	0.213	1180	1010	96.90	0.027	39.64	0.117	0.032	4.651	16.74	0.239	<LOD	196.2	0.460	47.80	69.83	38.22	3.707	0.949	11.36	7.538	0.012	4.852
43	CKLT1	Can	4.5	-	Cider	0.208	1357	1267	199.8	0.563	47.62	2.125	4.223	23.92	40.78	2.572	<LOD	438.2	0.859	25.53	564.0	71.40	113.5	12.75	150.3	2.123	0.021	224.9
44	SW2	Can	4.5	Citric acid	Other	0.046	1207	1026	71.60	0.296	33.63	0.153	0.268	2.258	8.346	0.625	<LOD	176.6	0.613	51.74	64.97	153.0	3.812	0.319	4.953	<LOD	0.019	9.025
45	CKWB	Bottle (White)	4.5	-	Cider	0.098	1135	880.8	84.70	0.342	58.45	0.182	7.568	16.21	8.962	0.317	<LOD	239.7	0.434	21.09	35.66	46.46	17.27	1.491	3.837	5.699	0.045	510.4
46	CKT	Bottle (White)	4.5	-	Cider	<LOD	1205	985.1	76.10	0.097	51.64	0.097	0.275	1.636	6.090	0.163	<LOD	237.3	0.089	21.09	31.26	46.17	1.581	1.034	3.266	0.652	0.019	8.687
47	RŻ2	Bottle (White)	4.5	Citric acid	Other	0.061	1314	1074	88.10	0.118	46.80	0.210	0.220	2.721	12.06	<LOD	<LOD	200.1	0.349	50.40	57.40	170.3	3.161	2.107	8.195	1.345	<LOD	2.521
48	RJTC2	Bottle (White)	4.5	Citric acid	Other	0.598	1393	1162	72.50	0.331	45.53	0.046	1.261	4.571	9.924	0.137	<LOD	174.4	0.274	48.24	41.61	21.61	5.256	0.244	4.881	<LOD	0.012	92.24

49	RMC	Can	4.5	Citric acid	Other	1.048	1383	1092	78.10	1.851	39.39	0.416	0.983	20.46	44.95	0.351	<LOD	137.7	0.702	39.85	110.2	39.09	16.91	52.87	18.78	<LOD	0.022	26.40
50	RMG2	Bottle (White)	4.5	Citric acid	Other	0.509	1905	1454	87.10	2.228	43.44	0.362	0.455	18.57	43.05	0.212	<LOD	177.0	1.046	47.49	146.2	26.89	15.31	21.14	18.92	<LOD	0.022	26.02
51	DWS	Bottle (White)	7	Citric acid	Other	0.575	1707	1190	89.00	3.526	50.67	0.294	0.610	29.76	95.38	0.528	<LOD	284.3	0.727	74.02	126.5	57.50	111.1	49.24	14.63	21.99	<LOD	35.97
52	DM	Bottle (White)	6	Citric acid	Other	1.126	1129	786.2	87.50	1.968	43.45	0.349	0.585	22.07	67.42	0.082	<LOD	255.5	0.753	58.61	104.5	50.39	22.93	13.91	13.19	24.02	<LOD	29.62
53	LFGA	Bottle (White)	0	Citric acid	Other	0.387	854.0	487.1	111.3	0.315	39.04	0.244	0.301	7.226	9.549	<LOD	<LOD	100.3	1.077	23.91	92.71	30.22	11.26	0.172	22.27	3.102	<LOD	8.772
54	LFMM	Bottle (White)	0	Citric acid	Other	2.569	904.0	477.3	117.8	1.150	36.76	0.396	0.680	7.708	4.937	<LOD	<LOD	101.7	0.839	22.25	68.99	43.37	12.38	<LOD	22.58	1.877	<LOD	18.24
55	LFLM	Bottle (White)	0	Citric acid	Other	0.462	995.0	500.7	129.6	1.084	35.61	0.379	0.402	11.34	11.65	<LOD	<LOD	103.0	0.775	21.52	73.49	42.22	12.93	16.94	23.64	4.963	0.011	9.004
56	CKLT2	Can	4.5	-	Cider	0.673	1293	1110	195.6	1.386	45.36	2.310	5.248	27.40	54.00	2.447	<LOD	739.8	1.167	39.77	649.5	108.6	152.5	17.05	168.8	6.300	0.190	267.0
57	CKLT3	Can	4.5	-	Cider	0.011	1020	1044	167.9	0.865	46.76	1.904	5.205	24.08	49.00	2.397	<LOD	753.7	0.995	40.86	582.4	118.5	145.8	12.68	160.7	7.800	0.099	256.0
58	CKLT4	Can	4.5	-	Cider	0.139	1231	1073	179.4	0.563	45.73	2.017	4.390	23.36	49.00	2.306	<LOD	746.6	0.747	39.78	571.8	105.3	142.1	13.53	157.9	4.600	0.077	316.0
59	CKLT5	Can	4.5	-	Cider	0.848	995.0	859.6	158.2	0.281	45.73	2.128	4.555	23.24	56.00	2.288	<LOD	748.9	0.702	40.29	581.0	105.3	141.5	13.80	157.2	5.700	0.055	338.0
60	CKLT6	Can	4.5	-	Cider	<LOD	1033	1364	85.10	0.186	33.97	3.074	3.257	19.56	44.00	1.735	<LOD	575.7	0.620	30.93	478.5	79.27	113.3	13.89	114.7	2.300	0.059	174.0
61	CKLB2	Bottle (Green)	4.5	-	Cider	0.465	1098	921.5	167.7	0.244	37.04	0.103	4.557	23.81	49.00	2.126	<LOD	689.5	0.880	36.69	507.5	111.1	143.8	14.80	125.4	8.500	0.062	257.0
62	CKLB3	Bottle (Green)	4.5	-	Cider	0.350	1117	1092	155.3	0.266	35.39	2.522	3.392	23.29	72.00	1.993	<LOD	647.7	0.901	29.67	337.2	126.9	127.7	12.61	116.9	3.500	0.137	265.0
63	CKLB4	Bottle (Green)	4.5	-	Cider	0.303	1269	1044	166.4	0.288	25.58	2.053	3.241	23.16	62.00	2.162	<LOD	649.8	0.777	30.04	347.6	106.1	125.9	15.59	106.3	6.400	0.065	355.0
64	CKLB5	Bottle (Green)	4.5	-	Cider	0.409	1324	1361	256.2	0.387	45.44	2.286	4.452	23.86	50.00	2.306	<LOD	757.9	0.845	40.84	577.3	130.8	130.6	13.71	157.0	4.800	0.509	290.0
65	CKLB6	Bottle (Green)	4.5	-	Cider	0.335	1059	989.1	195.8	0.252	44.03	1.536	4.378	24.17	66.00	2.117	<LOD	696.2	0.829	35.76	501.8	112.7	135.9	13.80	162.5	6.900	0.081	309.0
66	JR	-	6	-	Juice	0.649	1081	1041	465.2	0.392	120.4	1.634	7.868	22.31	122.0	2.428	<LOD	893.1	1.550	72.77	537.6	26.79	146.7	12.97	1147	5.000	0.088	314.0
67	CR1	-	6	-	Cider	0.472	590.0	603.3	433.7	0.522	124.7	2.239	10.85	24.42	97.00	2.717	<LOD	936.2	2.010	73.58	579.1	29.15	184.3	13.24	1217	15.30	0.127	277.0
68	CR2	-	6	-	Cider	0.150	417.0	935.8	460.1	1.256	127.9	2.006	9.066	24.91	104.0	9.090	<LOD	980.3	2.358	79.38	577.6	31.48	180.2	14.38	1203	15.90	0.124	436.0
69	CR3	-	6	-	Cider	0.120	605.0	544.4	422.8	0.868	122.0	1.951	11.53	24.17	115.0	2.963	<LOD	918.7	1.984	72.64	568.3	28.47	185.4	13.42	1194	18.80	0.080	403.0
70	JL	-	0	-	Juice	0.288	1326	1245	201.1	0.404	34.00	0.349	4.512	22.56	410.0	1.975	<LOD	1 053	1.224	47.74	353.0	16.71	170.7	18.72	51.48	4.100	0.097	606.0
71	CL1	-	8	-	Cider	0.118	500.0	486.7	111.0	0.914	37.70	1.751	4.382	25.17	364.0	2.689	<LOD	1 038	1.478	44.70	347.5	11.46	199.2	12.70	45.32	5.200	0.117	353.0
72	CL2	-	8	-	Cider	0.073	704.0	686.9	128.9	0.471	38.09	1.895	4.762	24.50	490.0	4.854	<LOD	1 050	1.390	45.66	341.3	13.62	192.1	12.82	48.80	6.700	0.061	494.0
73	CL3	-	8	-	Cider	0.163	917.0	876.8	157.2	0.415	37.97	1.902	4.964	27.33	358.0	2.411	<LOD	1 023	1.910	44.31	372.2	21.61	191.3	12.61	51.76	7.400	0.080	411.0
Limit of detection (LOD)						0.010 µg/L	-	-	-	0.010 µg/L	-	-	-	-	-	0.010 mg/L	0.23 ng	-	-	-	-	-	-	0.010 µg/L	-	0.042 µg/L	0.010 µg/L	-
